# The economic impact of the COVID-19 pandemic on ethnic minorities in Manchester: lessons from the early stage of the pandemic

**DOI:** 10.3389/fsoc.2023.1139258

**Published:** 2023-05-19

**Authors:** Arkadiusz Wiśniowski, Ruth Allen, Andrea Aparicio-Castro, Wendy Olsen, Maydul Islam

**Affiliations:** ^1^Social Statistics Department, The University of Manchester, Manchester, United Kingdom; ^2^Policy@Manchester, The University of Manchester, Manchester, United Kingdom

**Keywords:** ethnic minorities, COVID-19, UK local authorities, economic hardship, employment, Manchester

## Abstract

This review summarizes the economic impacts of the pandemic on ethnic minorities, focusing on the city of Manchester. It utilizes multiple reporting sources to explore various dimensions of the economic shock in the UK, linking this to studies of pre-COVID-19 economic and ethnic composition in Manchester and in the combined authority area of Greater Manchester. We then make inferences about the pandemic's short-term impact specific to the city region. Greater Manchester has seen some of the highest rates of COVID-19 and as a result faced particularly stringent “lockdown” regulations. Manchester is the sixth most deprived Local Authority in England, according to 2019 English Indices of Multiple Deprivation. As a consequence, many neighborhoods in the city were always going to be less resilient to the economic shock caused by the pandemic compared with other, less-deprived, areas. Particular challenges for Manchester include the high rates of poor health, low-paid work, low qualifications, poor housing conditions and overcrowding. Ethnic minority groups also faced disparities long before the onset of the pandemic. Within the UK, ethnic minorities were found to be most disadvantaged in terms of employment and housing–particularly in large urban areas containing traditional settlement areas for ethnic minorities. Further, all Black, Asian, and Minority ethnic (BAME) groups in Greater Manchester were less likely to be employed pre-pandemic compared with White people. For example, people of Pakistani and Bangladeshi ethnic backgrounds, especially women, have the lowest levels of employment in Greater Manchester. Finally, unprecedented cuts to public spending as a result of austerity have also disproportionately affected women of an ethnic minority background alongside disabled people, the young and those with no or low-level qualifications. This environment has created and sustained a multiplicative disadvantage for Manchester's ethnic minority residents through the course of the COVID-19 pandemic.

## 1. Introduction

Whilst the COVID-19 pandemic has undoubtedly affected lives universally, early decrees that the pandemic would be “a great leveler” (Milne, 2020) were imprudent. Across geographies and between individuals, the impacts of the pandemic have been asymmetrically distributed.

**Figure 1:**
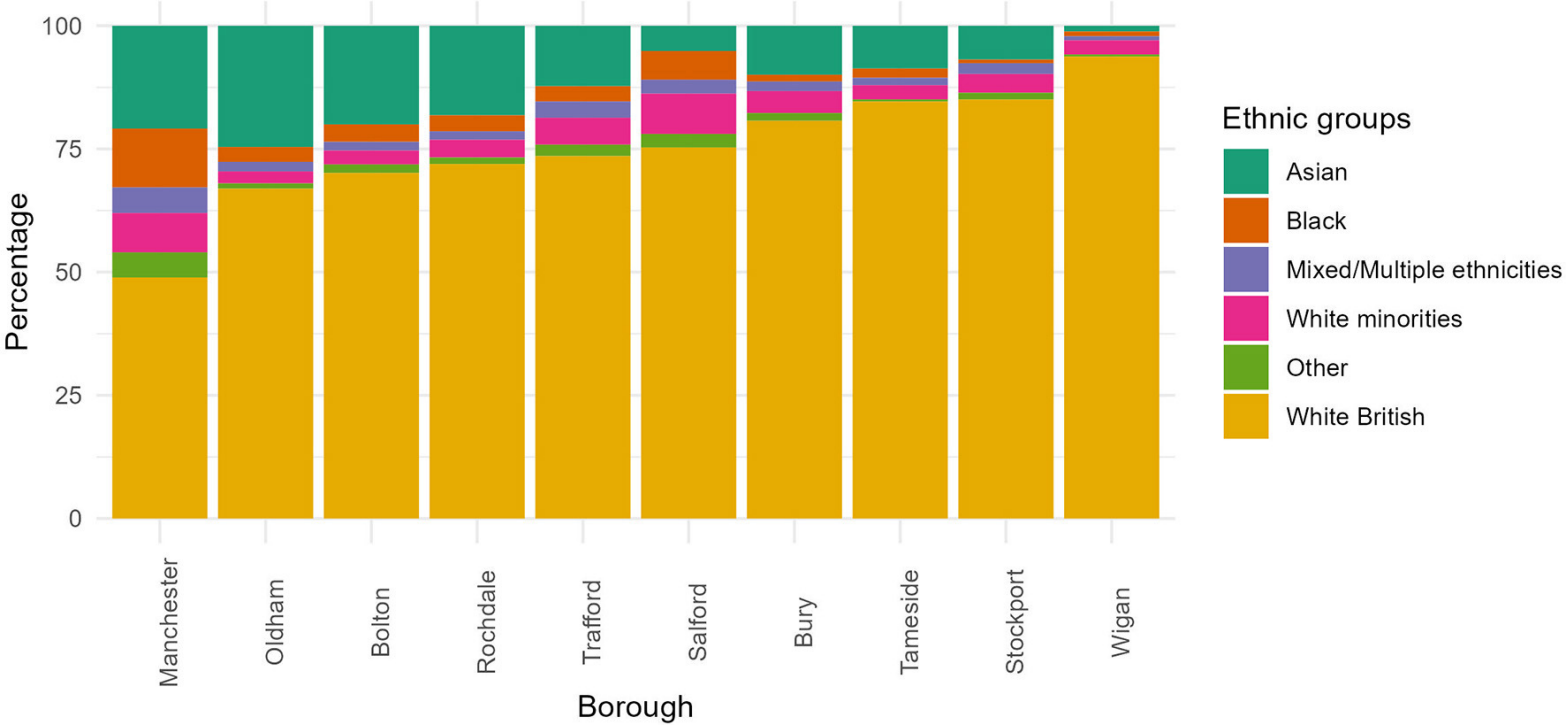
Percentage of population by broad ethnic groups based on the 2021 UK census categories in Greater Manchester boroughs, including the General Population (i.e., White British). The borough with the highest percentage of ethnic minorities is in the extreme left and the borough with the smallest percentage of ethnic minorities is in the extreme right. **White British** correspond to the General Population composed of White English, Welsh, Scottish, Northern Irish, and British. The **Asian** group is composed of Bangladeshi, Chinese, Indian, Pakistani, and Other Asian. The **Black** ethnic group includes African, Caribbean, Other Black. The **Mixed** or **Multiple** ethnic groups refer to White and Asian, White and Black African, White and Black Caribbean, Other mixed or multiple ethnicities. **White minorities** are composed of Gypsy or Irish Traveler, Irish, Roma, Other White. **Other** ethnic group category involves Arab and any other ethnicity. Source: UK 2021 Census.

This rapid review aims to summarize the economic impacts of the pandemic on ethnic minorities, with a focus on the city of Manchester. It utilizes multiple articles and reports to explore various dimensions of the economic shock in the UK, linking this to studies of pre-COVID-19 economic and ethnic composition in Manchester and in the combined authority area of Greater Manchester, in order to infer the pandemic's impact specific to the city region. The Greater Manchester is a combined authority made up of the 10 Greater Manchester councils (Bolton, Bury, Manchester, Oldham, Rochdale, Salford, Stockport, Tameside, Trafford and Wigan). Mid-2020, Greater Manchester had a population of 2.84 million, with 555 thousand people living in Manchester local authority (ONS, 2021a). Since 1991, the region's ethnic mix changed dramatically. For example, between 1991 and 2011, the ethnic minority population increased by more than 100,000 people, or 164% (Jivraj, 2013). In Manchester, the share of ethnic minority people (including non-British White) increased from 40% to more than 50% between 2011 and 2021 censuses (*idem*.; see Figure 1); the share of ethnic minorities is also considerably larger than in other boroughs of Greater Manchester. The largest ethnic population in Manchester is Asian (41% of all ethnic minorities), followed by Black people (23%) and Non-British White (nearly 16%); see Supplementary Figures 5, 6 for further details.

At the time of preparing this report, Greater Manchester had seen some of the highest rates of COVID-19 and as a result has faced particularly stringent lockdown regulations, with most of Greater Manchester's Middle Layer Super Output Areas (MSOAs)^1^ experiencing less than a month without enhanced restrictions since the pandemic began (Timms et al., 2020).

In this report, we use the term “ethnic minorities” to denote all ethnic groups other than White British.^2^ That is, ethnic minorities include Other White and Roma, Gypsy and Irish Travelers. The BAME category includes Black, Asian and minority ethnic groups. The use of this category is discouraged as it is problematic due to its unclear definitions and its focus on skin color (Khunti et al., 2020a). The BAME acronym is widely considered to mean “Black, Asian and minority ethnic” groups but can be misunderstood as “Black and Asian minority” ethnic groups, whereas the UK Equalities Act 2010 is clear in defining the protected categories including race and this Act is a key reference point. The UK government stopped using the “BAME” term as of March 2022 and recommended referring to ethnic minority groups individually.^3^ Where possible, we refer to specific minorities rather than to broader categories.

This review is structured as follows. In the rest of this section, we survey the data sources for analyzing the economic impacts of the pandemic on ethnic minorities, covering the situation before the COVID-19 pandemic, and health-related outcomes in the area. Section 2 discusses the findings in the literature that focused on employment, people's financial situation, food security and homelessness during the pandemic and can be used to infer the impacts on ethnic minorities. Section 3 discusses the findings and concludes.

### 1.1. Data availability

Interventions to mitigate the economic effects of the pandemic and the lockdown, and their evaluation, require timely, frequent and precise data on the economic and financial situation. These data should be available for small geographies (such as Local Authority^4^ or smaller) and disaggregated by key characteristics, such as age, sex, and ethnicity. Some data sources are available at small area geographies. For example, the Claimant Count and Flow data are the counts of Universal Credit and Job Seekers Allowance claimants broken down by sex, age and type of benefit claimed (see Supplementary material 1 and Section 2.2). They are available monthly via the Nomis^5^ database for Lower Layer Super Output Areas (LSOAs) but without information on the ethnicity of claimants.

Other measures of economic impacts of the pandemic, disaggregated by ethnicities and small geographies, are available from surveys, such as Labor Force Survey/Annual Population Survey (LFS/APS), or Understanding Society UK Household Longitudinal Study (see Supplementary material section 1 for a brief overview). Even though many of these surveys were promptly adopted to collect data via remote modes rather than face-to-face interviews, their results typically came with delays, which hinders design of timely interventions, and suffers from sampling and non-sampling errors. The non-response and attrition further exacerbates analytical problems where detailed information is required to make inferences about ethnic minorities, further intersected with other relevant characteristics, such as smaller levels of geography (e.g. the city of Manchester, LSOAs), gender, occupation, migrant status, and other. This can lead to studies that use the same data yielding incompatible conclusions (see, e.g., Section 2.1.2).

Therefore, this review focuses on findings related to ethnic minorities on national and regional scales, as well as pre-pandemic analyses that used small geographies. Where available, we refer to the data for Manchester, as well as for other geographies, to illustrate the situation of ethnic minorities in the period immediately preceding the pandemic. This review is of use to future researchers on the UK who can triangulate or combine datasets.

### 1.2. Manchester pre-pandemic

#### 1.2.1. Income and deprivation

Manchester, along with a further three of Greater Manchester's Districts (Oldham, Rochdale and Salford), appeared in a table of 20 local authorities (LAs) with the highest proportion of LSOAs in the most deprived 10% nationally on the Index of Multiple Deprivation (IMD) in 2019 (Penney, 2019). Further, ONS data on Gross Disposable Household Income (GDHI; calculated per head of population at current basic prices) for 2018 also showed that Manchester had a third largest average annual growth of GDHI (ONS, 2020d). Nevertheless, it was the poorest Local Authority in Greater Manchester city-region, averaging household incomes of £14,864, followed by Oldham and Rochdale (*idem*.).

Four of the six areas with the lowest average household incomes (Manchester, alongside Bolton, Rochdale and Oldham) contained wards with ethnic minority populations making up 20–60% of the population (Jivraj, 2013). They also had between 24 and 43% of their respective LSOAs ranked as in the most deprived 10% in England (Penney, 2019). Within the UK, ethnic minority groups were found to be most disadvantaged in terms of employment and housing, particularly in large urban areas containing traditional settlement areas for ethnic minorities (Lymperopoulou and Finney, 2017; Clark and Shankley, 2020; Shankley and Finney, 2020). This was reflected in the Greater Manchester's pre-pandemic labor market where all ethnic minority groups were less likely to be employed than White people with Pakistani and Bangladeshi people, especially women, having the lowest levels of employment (Elahi, 2017).

#### 1.2.2. Employment

Investigating the economic resilience of UK regions in the context of the recovery from the 2007/8 global financial crisis, Sensier and Devine (2020) noted that Greater Manchester scored slightly higher for resilience than the rest of the North West due to its strong employment growth, despite subdued productivity growth. However, Hincks' (2017) analysis of deprived neighborhoods across Greater Manchester found that the most deprived neighborhoods were subject to higher volatility in response to economic shocks when compared with less-deprived neighborhoods. Further, Hughes and Lupton (2018) found that deprived neighborhoods in Greater Manchester were undergoing changes in opportunities for income, housing, and employment as well as migration and ethnic composition. However, low qualifications, poor health and caring commitments were all found to be much more salient issues in most deprived neighborhoods of the region than changes in patterns of residential mobility.

Lupton et al. (2016) highlighted low paid work as a substantial issue for the labor market in Greater Manchester. In their report on inclusive growth in the area, they noted that in 2015, 23.2% of jobs in Greater Manchester paid less than the UK Living Wage.^6^ Similarly, Jeffery et al. (2018), in their qualitative study of work and welfare in Salford, considered low paid and precarious work and the introduction of Universal Credit, with its system of claimant commitments that jobseekers had to adhere to, and punitive measures if conditions were unmet (p. 797–798), to be detrimental to equal opportunities in the area. The authors drew attention to the polarization in occupational growth, noting that increases in the lowest ranked occupations from 12.6% in 2004 to 17.1% of the working-age population's primary jobs in 2016 are in stark contrast to the simultaneous increases in “managerial, professional, [and] technical” sectors, which in 2016 accounted for 37.3% of all jobs in Salford (Jeffery et al., 2018). However, this increase can be attributed to the jobs created by the Media City project at Salford Quays, a building programme developed over 2007–2011.

A skills analysis of Greater Manchester by New Economy (2017) found that information and communication jobs topped the list of growing employment sectors in the area. However, overall, jobs in Greater Manchester were lower skilled than is typical in the UK, with particularly high concentrations of low-skilled workers in Manchester, Oldham, Rochdale, Bolton and Wigan. These areas also have higher than average numbers of deprived Lower Super Output Areas (LSOAs), and four of these boroughs have high percentage of ethnic minority populations overall; see Table 1.

**Table 1:** Universal Credit count as a percentage of residents aged 16–64; March and July 2020, Greater Manchester boroughs (Manchester in bold).

	**Bolton**	**Bury**	**Manchester**	**Oldham**	**Rochdale**	**Salford**	**Stockport**	**Tameside**	**Trafford**	**Wigan**
March 2020	4.9	3.7	**4.5**	5.1	4.7	4.2	2.9	4.2	2.5	3.9
July 2020	8.4	7.1	**8.7**	9.4	8.6	8.1	6.0	7.9	5.3	6.9
Increase in p.p.	3.5	3.4	**4.2**	4.3	3.9	3.9	3.1	3.7	2.8	3.0
% of ethnic minority in total 16+ population	20.0	11.9	**32.4**	21.6	21.3	13.1	8.0	9.0	15.7	4.6

Source: percentage of ethnic minority in the population based on Annual Population Survey and claimant count retrieved from Nomis database.

#### 1.2.3. Housing

As of 31 March 2020, there were 15,689 Local Authority-owned dwellings in Manchester, almost all of them were rented socially. The stock of social housing remained at similar levels as in previous years (DLUHC, 2020). Meanwhile, the English Housing Survey 2016–18 (Ministry of Housing, Communities and Local Government, 2020a) revealed that, in England, the percentages of households that rented social housing were highest for Black African (44%), Mixed White/Black African (41%), Black Caribbean (40%) and Bangladeshi (33%), as compared with 16% for the White British. North West also had the second largest (after London) proportion of people “Other than British White” renting social housing (21%), as compared with White British (17%) (*idem*.).

Further, the English Housing Survey (2021) also showed that in 2019/20, 9% of houses in the social renting sector were overcrowded, the highest levels since 1995/96. The rate of overcrowding was highest amongst local authority tenants (11%), as compared with housing association tenants (7%).

#### 1.2.4. Inequalities, deprivation and policies

The inequalities found in Greater Manchester's labor market pre-COVID-19 reflect the structural inequalities at a national level in form of social and economic inequalities underpinned by institutional racism and reduced access to resources (see, e.g., Nazroo, 2003; Bentley, 2020; Stopforth et al., 2022), especially for older ethnic minority people (Bécares et al., 2020). Manchester's residents are also impacted by interregional inequality. Prior to the onset of the pandemic, the UK was found to be one of the most unbalanced countries in the industrialized world in terms of interregional inequality, when assessed by multiple measures including Gross Domestic Product (GDP) per capita, regional Gross Value Added (GVA) per capita and Regional Disposable Income (RDI) per capita (McCann, 2020). Inequality in the UK was more apparent in regional comparisons than in those between urban and non-urban areas (McCann, 2016). There was a clear North-South divide, with 15 out of the 20 most deprived areas in the UK in 2019 being in the North or Midlands, according to the IMD (Penney, 2019).

These most deprived areas had historically not felt the rewards of globalization and national economic growth, fuelling a feeling of being “left behind” which many cite as a primary reason behind the result of the EU Referendum in 2016 (Goodwin and Heath, 2016). The government has faced criticism for this disparity for decades. To enable local authorities to address the issues facing their communities, in 2021 the UK government committed to a “leveling up” agenda, with a fund of £4.8bn focused on the local infrastructure in the regions “left behind” as a part of a broader package of the UK-wide interventions.^7^ However, prior to that, Etherington and Jones (2017) provided a detailed analysis of Greater Manchester's devolution strategy, arguing that unprecedented cuts to public spending because of austerity^8^ undermined the extra funding given for the City Deal^9^. Comparing local authority funding from 2009/10 to 2016/17, they note budget gaps pointing to a loss of over £1bn. They stress that women, those of BAME ethnic background, disabled people, young people and those with no or low-level qualifications have been disproportionately affected by these cuts (cf. Sandhu and Stephenson, 2015; Hall et al., 2017).

As ONS reported, despite the redistributive power of taxes and benefits, the gap between the richest and poorest income in the UK has widened over last 10 years (ONS, 2021b). The rise in inequality can also be attributed to the freeze on cash benefits in the financial year ending 2016 (*idem*.).

In summary, it is evident that those of ethnic minority background, as well as women and young people, faced disparities long before the onset of the pandemic. As Manchester is a relatively deprived area, ethnic minority people within Greater Manchester, and in Manchester specifically, face more deprivation and are in precarious situations. It is this environment that created and sustained multiplicative disadvantage throughout the course of the COVID-19 pandemic.

### 1.3. COVID-19 transmission and mortality amongst ethnic minority population

Greater Manchester had identifiable health inequalities prior to the pandemic, with one of the lowest life expectancies in England for both men and women (life expectancy at birth for males in 2017–19 was 76.4 and for females 80.3, which are 3.4 and 3.1 years lower than respective values for England, ONS, 2020f). That the North West had the highest percentage of its population shielding in the UK, at almost 5% of the population (Marszalek and Peytrignet, 2020), is therefore unsurprising. The links between socio-economic and health deprivation are clear (see, e.g., Nazroo, 2003; Bambra et al., 2011, 2020b; Marmot, 2020).

Ethnic minorities were at a higher risk of infection, mainly because they tend to live in urban and deprived areas, overcrowded households, and work in occupations with higher risks of transmission (e.g., Nazroo and Becares, 2020). They may also be born abroad and thus face cultural and language barriers in accessing services (Public Health England, 2020a). Further, some ethnic minorities may be at a higher risk of poor outcomes once they acquire the infection due to the higher rates of pre-existing conditions, for example, the prevalence of hypertension was significantly higher for Afro-Caribbean and South Asian populations (Khan and Beevers, 2005; Public Health England, 2020a). It is worth noting that, at the early stage of the pandemic, racializing assumptions that susceptibility to COVID-19 lies in genes (El-Khatib et al., 2020; Khunti et al., 2020b) were soon rejected and the focus shifted toward social and structural differences between ethnicities and comorbidities (Bentley, 2020; El-Khatib et al., 2020).

The ONS data (ONS, 2020e) showed that, in England, males of Bangladeshi and Black African ethnicity had the risk of death from COVID-19 almost twice as large as the White population, after controlling for age, type of residence, geography, socio-economic status, household characteristics, occupational exposure, and health-related variables. For females in the same ethnic groups, the odds ratios were 1.51 and 1.34, respectively, meaning that the risk of death was one and a half and 1.34 times larger for them, compared with the White population. There were also differences in how ethnic minorities were affected over time. In the first wave,^10^ risk of mortality was the highest for Black African (both males and females), whereas in the second wave, it was for Bangladeshi, Pakistani and Indian minorities (*idem*.). Public Health England (2020a,b), by using the data from the ONS, also reported large disparities in infection and mortality rates for BAME individuals across England. Public Health England (2020a) suggested that death rates among those known to have had COVID-19 were twice as high for people of a Bangladeshi background and 10–50% higher among other ethnic groups compared to White British people, after controlling for age, sex, deprivation and region. The PHE's report also found that Asian and Other ethnic groups were over-represented in the number of COVID-19 cases amongst nurses, midwives and nursing associates, as opposed to White and Black ethnic groups. Occupations that experienced significantly higher mortality than the rest of the population aged 20–64 were nursing auxiliaries and assistants, security guards and related occupations, and taxi and cab drivers and chauffeurs; however there was no breakdown of mortality by occupation and ethnicity (see also Williamson et al., 2020). Patel P. et al. (2020) criticized this PHE report for not including discussion and recognition of the structural inequalities which are fuelling the pandemic's disparate effects (see also Bentley, 2020).

These findings were in line with Prats-Uribe et al. (2020) who considered socioeconomic deprivation and prior comorbidities as factors behind ethnic minority communities' higher rates of infection. Their findings indicated that Asian/Asian British and “Other” ethnic group participants had around double the infection risk of White participants. The UK Biobank data they used slightly underrepresents ethnic minorities and people from socioeconomically deprived areas (Fry et al., 2017), but low representativeness does not imply which direction any bias in the risk estimate might go.

Further, a report from the Royal College of Nursing (2020) suggested that BAME health workers have been disadvantaged in access to PPE compared to their White counterparts. Meanwhile, a survey of 200 Asian doctors and nurses in Leicester in May 2020 found that more than 70% were anxious about their role (Moorthy and Sankar, 2020). A survey of almost 1,000 health and social care staff found that 50% felt their general mental health had declined during the first 2 months of the pandemic (Thomas and Quilter-Pinner, 2020). However, that study did not collect data regarding the ethnicity of respondents, leaving questions regarding racial/ethnic differences in the mental wellbeing of healthcare staff unanswered.

Several studies (Chandola et al., 2020; Pierce et al., 2020; Proto and Quintana-Domeque, 2021) also demonstrated the effects of the first lockdown on mental health in the UK. Proto and Quintana-Domeque (2021) discovered that BAME men, and both BAME and British White women experienced larger deteriorations in mental health than British White men. They also ascertained that Bangladeshi, Indian and Pakistani respondents drove the gap between mental health outcomes for BAME and White men.

In May 2020, Patel J. A. et al. (2020) urged policymakers to expand their definition of the vulnerable to include those with socioeconomic vulnerabilities, not only those with multiple comorbidities for the virus. They identified six factors putting those of low socio-economic status (SES) at higher risk for exposure to COVID-19: (1) a higher likelihood of living in overcrowded housing; (2) employment in occupations with no scope to work from home; (3) unstable work conditions and incomes which affect mental health; (4) that those of lower SES present to healthcare at more advanced stages of illness; (5) barriers to accessing healthcare services, including language barriers; (6) hypertension and diabetes as more common in poverty-stricken populations and ethnic groups (Khan and Beevers, 2005). Clift et al. (2020) developed a so-called QCOVID-a prediction algorithm for the risk of hospital admission and mortality due to COVID-19. It takes into account, amongst other predictors, ethnicity and a Townsend deprivation score^11^ based on a patient's postcode. Finally, Nazroo and Becares (2020) and Razai et al. (2021) asserted that a discussion of ethnic disparities in COVID-19 should also to consider the effect of cultural and structural racism.

Several studies investigated COVID-19 mortality rates across England and Wales, finding evidence that they are consistent with the geography of economic deprivation and ethnic composition. Areas with greater than average concentrations of non-White populations experienced a five-percentage-point higher death toll (Tubadji et al., 2020; see also Nazroo and Becares, 2020; Williamson et al., 2020; Breen and Ermisch, 2021). In particular, Williamson et al. (2020) found that Black and South Asian people have been at higher risk of dying compared with those of White ethnicity. Sá (2020) conducted a similar geographical analysis, adding that areas with large households and greater use of public transport also have higher infection rates.

Breen and Ermisch (2021) similarly examined geographical variation in age and sex-standardized COVID-19 mortality rates across English local authorities between March and July 2020 by using ecological models. Although areas with higher social deprivation had higher COVID-19 mortality rates, this association was not as strong as that between social deprivation and total mortality rate, whilst an area's non-White proportion of population was found to be strongly associated with COVID-19 mortality. This suggests there were differences in the relationship between socioeconomic factors and COVID-19 mortality when compared with non-COVID and past mortality. Nazroo and Becares (2020) also found that rates of COVID-19-related mortality within a local authority were higher when the proportion of ethnic minority population was higher. They explained that the reason for this are social and economic inequalities driven by entrenched structural and institutional racism and racial discrimination (Bentley, 2020).

## 2. Impacts of COVID-19 restrictions and lockdowns on the economy

The impact of the pandemic on the global economy was documented over its course, with the shock from lockdown evident across various measures of economic outcomes (e.g., Baker et al., 2020; Maital and Barzani, 2020; Nicola et al., 2020). While the ramifications of the pandemic may be ubiquitous, the distribution of these consequences was not. Those more likely to be exposed to financial stressors such as income poverty, food poverty, and insecure employment pre-pandemic, were also more likely to have faced disproportionate impacts on their financial wellbeing as a result of the lockdown. Negative economic impacts were also shown in the absence of the restrictions, for example, in South Korea (Aum et al., 2020).

As the economic response to the pandemic has differed by country, so too have the economic impacts. Adams-Prassl et al. (2020) presented survey evidence from the UK, US and Germany, illustrating differential labor market impacts across the countries. The analysis evidenced that respondents in Germany had faced much less of an impact on employment than their UK and US counterparts. For example, just 5% of German workers had lost their jobs in early April 2020, compared to 20% in the US and 17% in the UK. Further, female workers were more affected by job losses in the UK and US, but not in Germany–the authors attribute this to differences in care responsibilities. Fana et al. (2020) analyzed employment across six European countries using EU-LFS data. They suggested that countries affected in the early stage of the COVID-19 pandemic in terms of the numbers of contagions and deaths (Spain, Italy and the UK) were most likely to suffer the worst implications of lockdown due to having a larger share of the workforce employed in shut down sectors, such as hospitality and retail; accommodation, leisure and tourism; and personal care activities. The authors drew attention to the fact that these labor markets were most vulnerable prior to the onset of the pandemic, “characterized by high unemployment and precarious work.” Also, ethnic minorities were likely to be disproportionately represented in these sectors (Citizen's Advice, 2020; see also Sections 2.1.1 and 2.1.2).

Susskind and Vines (2020) recount the economic milestones of the pandemic and summarize the global changes in policy that were put in place to counter the extreme impacts of the pandemic. In the UK, this included the following: (i) The Coronavirus Job Retention Scheme, which allowed any employer in the UK to apply for a government grant to cover 80% of the wages (up to a total of £2500 per month) of employees who were not working but were “furloughed” and kept on payroll, rather than being dismissed (HM Treasury, 2020). (ii) The Self-Employment Income Support Scheme, which allowed self-employed individuals or partnerships to apply for a grant equalling 80% of 3 month's average trading profits, paid in a single installment (capped at £7,500 in total) per quarter (HMRC, 2020). (iii) An increase of £20 per week to the standard allowance in Universal Credit and the basic element in Working Tax Credit (DWP, 2020). This uplift was ended in October 2021. (iv) Since 28 September 2020, the UK government's regulations^12^ have required those tested positive or had symptoms of COVID-19 and those in their households, as well as those notified by the National Health Services (NHS) Test and Trace, to self-isolate for 10 days. This regulation prompted the introduction of the Test and Trace Support Payment scheme in form of a £500 lump sum paid to those on low incomes. The government estimated just below four million people would be eligible for this payment (Kennedy and Ferguson, 2021). However, as TUC (2021) showed, the available funding fell short of the demand, with seven out of 10 applicants left without support and the estimated cost being more than twice the allocated budget. (v) In March 2020, the government banned bailiff enforcement of evictions (“Eviction Ban”) and no new possessions were to be started during the crisis (Ministry of Housing, Communities and Local Government, 2020b).

Regarding northern cities of England, Bambra et al. (2020a) analyzed the effect of COVID-19 on productivity, mental and financial wellbeing, economic crises, health, and children. They stated that the North-South divide and resulting ability of the North to withstand the economic impacts of the pandemic puts the government's “leveling up” project (see Section 1.2.4) in immediate danger, with prior inequalities between regions exacerbated by the pandemic. Detailed description of various economic impacts of the pandemic in Manchester and the region is presented below.

### 2.1. Changes in employment circumstances

The data compiled by the ONS (2020c) data showed that UK's Black youths (aged 16–24) experienced the largest unemployment rate throughout 2020, compared with the White population. For example, in October-December 2020, this rate was 41.6% for Black youths compared with 12.4% for White, while in the respective quarter in 2019, the rates were 24.5% and 10.1% for Black and White, respectively. The second most affected group of youths were Pakistani (31.5% in Oct-Dec 2020, compared with 22.5% in Oct-Dec 2019). The unemployment rate in Oct-Dec 2020 for all over 16 s was the highest amongst Pakistani individuals, at 9.7%, compared with 4.5% for White and 7.6% for Black population.

Meanwhile, the unemployment rate in Manchester increased from 4.9% in 2018 (January to December), through 6.2% in 2019, to 8.7% in 2020. For comparison, the UK rates in these years were 4.2, 3.9, and 4.6%, respectively. Further, 18.3% of all households in Manchester were jobless, compared with 16.2% in Greater Manchester, and 13.3% in England (Annual Population Survey, 2021). Because estimates are based on the LFS/APS, the ones for Manchester come with much larger uncertainty; e.g., the 95% confidence interval for 2020 unemployment rate was (6.0%, 11.4%) for Manchester and (4.5%, 4.7%) for the UK.

As 2021 Census data showed,^13^ in Manchester, White British had the largest proportion of people in managerial occupations (9.7% of all White British), compared with 3.9% of Black (see Supplementary Figure 1). Black people were over-represented in caring, leisure and other service occupations, potentially increasing risks of being exposed to COVID-19 as frontline workers (see Section 2.2). Black people also had the largest proportion of the population in elementary occupations (23.5%), followed by Other ethnic groups (16.6%); White British had the lowest proportion of 11.1%.

This is in line with the review of policies and predictions on the ability of the UK economy to recover post-COVID-19 (Mayhew and Anand, 2020). The authors predicted that the young would suffer disproportionately from a COVID-19-triggered recession, warning against policies that prioritize getting people into work over matching people to the right jobs, which have inadvertently contributed to unemployment scarring in the past. Work-based training and apprenticeships, and better training of work coaches were seen as solutions to this issue.

#### 2.1.1. Self-employed workers

Data from the APS showed that, in Manchester, in 2018 (January to December), 12.3% were self-employed, compared with 15.0% in the UK. In 2020, this value fell to 14.0% in the UK and to 8.7% in Manchester (Annual Population Survey, 2021). However, it is important to note that there were considerable differences in self-employment by ethnic group. For instance, one in four Pakistani and one in five Bangladeshi people of working age were self-employed in England and Wales (Lawrence, 2020; Platt and Warwick, 2020). In 2018, self-employment in the UK was most common in the combined Pakistani and Bangladeshi ethnic group, where 20.4% of workers were self-employed, while for the White people this percentage was 15.1%. Further, 41% of workers from these two ethnic groups were in the three least skilled types of occupation, as compared with 24% for the White.^14^ Therefore, these ethnic groups were disproportionately exposed to the aforementioned ramifications of COVID-19 on the self-employed. This is of particular concern as Greater Manchester is home to large Pakistani and Bangladeshi communities, with large populations of these ethnic groups residing in Manchester as well as Rochdale and Oldham (ONS, 2012).

The UK LFS data were used by Reuschke et al. (2020) who found, during the first lockdown, large declines in self-employment to levels previously seen in 2016 and that the decreases affected men proportionately more than that of self-employed women. Analysis included numbers and change in self-employment by region (London saw the largest decline during the period) as well as ethnic groups. Black African, Black Caribbean and Black British people had the largest decreases in self-employment, with these groups seeing a reduction of two percentage points in April-June 2020, compared to the same period in 2019. As mentioned before, the LFS has too small sample sizes for a meaningful analysis of outcome for specific ethnic groups in Manchester.

Further investigation into self-employment during 2020 using survey data collected by the London School of Economics and the Center for Economic Performance (Blundell and Machin, 2020; sample size around 1,500) revealed that although self-employed men's work declined more than women's, this is due to self-employed women being able to work from home more frequently than self-employed men. For those able to work from home, women were more negatively affected than men. The authors also noted that higher-income workers were more likely to apply for the Coronavirus Self-employment Income Support Scheme. These findings were mirrored in evidence from one business owner interviewed in Lawrence's (2020) review of the disproportionate impact of COVID-19 on ethnic minority communities, who conducted a survey that showed 48% of businesses led by ethnic minority people did not apply for government schemes because they did not think they would qualify.

Yue and Cowling (2021) used the first sweep of the COVID-19 Understanding Society survey (ISER, 2021) and found disproportionate effects on subjective wellbeing for the self-employed when compared with waged workers, linking this to the asymmetric government response in financial aid, which favored waged employees and left around one million self-employed workers with no financial support (Lawrence, 2020). The authors also highlighted a difference in the impact of a reduction in work hours on the wellbeing of the self-employed, indicating that they were attached to their work in a way that “transcends that of waged employees” (Yue and Cowling, 2021, p. 107).

#### 2.1.2. Employees

Nearly 50% of the jobs at risk during the first lockdown were in occupations paying < £10 per hour (Allas et al., 2020; analysis based on Business Impacts of COVID-19 Survey). They also reported that 73% of those working in accommodation and food services were furloughed in the first half of April, whilst only 13% of those in information and communication experienced the same fate. The median gross hourly pay in these sectors in 2019 was £8.60 and £19.20 respectively (Allas et al., 2020). The authors also found that regions with lower average income had a higher proportion of jobs at risk.

Crossley et al.'s (2021) study of labor market shocks and households' coping strategies with COVID-19 Understanding Society survey data (ISER, 2021) found that although 45% of individuals had experienced a loss of household earnings of at least 10%, those from minority ethnic groups and those in the lowest two quintiles of average pre-COVID income suffered the worst labor market impacts. For those whose hours fell, BAME groups were 15 percentage points (p.p.) less likely to be supported by the Job Retention Scheme, and 13 p.p. more likely to have been made unemployed as a result of the pandemic. The BAME category was used due to explicitly acknowledged small sample sizes. The median fall in earnings for the bottom quintile of earners was 13% by May, versus 2% for the top quintile. These groups also had higher rates of additional borrowing in order to mitigate these impacts. However, the above findings contrast with Witteveen's (2020) analysis that relied on the same data. Witteveen (2020) indicated that women were less likely to have been furloughed or dismissed from work, attributable to the fact that these groups were more frequently employed in essential occupations. For the ethnic minorities (again classified as BAME), Witteveen (2020) found no difference when compared to the White population, except for the middle tertile of earnings. The likely explanation was again the fact that ethnic minorities were more likely to be employed in essential occupations. The analysis did not test for possible moderation of the effect of being a BAME person with the type of employment or gender. In both analyses, the sample sizes were relatively small, with around 6% of the samples being BAME people (as compared with an estimated 14.4% for the UK). Crossley et al. (2021), however, used model-calibrated inverse-probability weights. The differences in substantive findings between the two analyses demonstrate the difficulty in providing policy recommendations based on the survey data with relatively small sample sizes of ethnic minority population, even at a national level.

Crawford et al. (2020), by using the 2017 ONS Living Costs and Food Survey, showed that pre-pandemic, households in the top quintile of income spent around a third of their expenditure on services affected by sector shutdowns, including transportation and hospitality and leisure, for example, personal care, restaurants, and culture. Blundell et al. (2020) make the salient point that these sector shutdowns removed the livelihoods of many lower earners (based on Family Resources Survey). Findings from the Resolution Foundation's coronavirus survey (with 6,000 respondents) largely confirmed that those with the lowest earnings borne the brunt of the economic impact of the pandemic (Gardiner and Slaughter, 2020). The authors found that around a third of lower-paid employees had lost their job or been furloughed as of May 2020, in comparison to around a tenth of top earners. Employees in atypical work, such as those on zero-hours contracts, were also more likely to have experienced job or hours losses or been furloughed.

This is confirmed with analysis from Citizen's Advice (2020), based on a survey with around 6,000 respondents, which finds that those in insecure work were 1.5 times more likely to have been made redundant than other working-age adults, and seven times more likely to have had informal redundancy discussions. People from ethnic minority backgrounds represented a disproportionate percentage of the insecure workforce, with 18% of all insecure workers being classified at that time as BAME, despite these groups making up 11% of the total workforce (Citizen's Advice, 2020). In particular, Black people were over twice as likely to be in temporary work, and the percentage of the Black workforce on zero-hour contracts was almost double the UK's average (TUC, 2017).

Platt and Warwick (2020) assessed differential impacts of the pandemic between specific ethnic minority groups. The authors used data from the LFS. They found substantial variability between the major ethnic minority groups, with White Other and Indians being more comparable to White British in terms of economic vulnerability. In contrast, Bangladeshi and Pakistani men were at particular risks of earnings losses due to a prevalence of working in self-employment and shutdown sectors coinciding with high occurrence of single-earner households in these groups.

Blundell et al. (2020) similarly highlighted Pakistani and Bangladeshi as being vulnerable to job and earning losses because they constituted 30% of workers in shut-down sectors. Key workers were also disproportionately represented among ethnic minorities, especially Black people, who, thus, faced disproportionate exposure to the virus.

#### 2.1.3. Job search

The pandemic's impact on the jobs market in the UK was assessed by Arthur (2021) with data from the online job platform Reed.co.uk. Results showed that although job postings rapidly fell in March 2020, they subsequently slowly increased. By January 2021, the number of posts per day had recovered to pre-crisis levels. The pattern of job adverts per capita was consistent across the UK's NUTS2 regions. Even despite the North West being the worst affected by COVID-19, the pattern of job postings was largely comparable to that of London. However, a clear North/South divide was present, with the number of advertisements by population showing a concentration in the South East. The authors noted, however, the limitations of the data in analyzing regional patterns.

In Manchester, advertised vacancies halved between May 2020 and July 2020 (THINK, 2020). Increases in the numbers claiming unemployment benefits in Manchester neighborhoods with high proportions of ethnic minority populations were reported to be a result of many residents in these areas losing work due to the closure of the night-time economy and hospitality sectors (THINK, 2020; see also Case Study in Supplementary material section 2). Increases in Universal Credit claims coincided with temporal suspending of the work-search requirements and attending regular interviews at jobcentres. Some of these crisis measures started to be withdrawn as soon as summer 2020 (Mackley, 2021).

#### 2.1.4. Flexible working arrangements

A report by Yasenov (2020) on United States data highlighted a barrier to financial resilience during the pandemic, that is, those with socioeconomic vulnerabilities were less likely to be able to work from home, including those with lower levels of education, ethnic minorities, lower-wage workers, and immigrants. Interestingly, the ONS's (2020b) Coronavirus and Homeworking report, based on the prototype ONS Labor Market Survey, found that, in the UK in April 2020, 48.1% of people from ethnic minorities reported some homeworking, compared to 46.4% of White people. Of those in ethnic minority groups who worked from home, 87.3% said that COVID-19 was the main reason for homeworking, compared with 85.8% for the White respondents. However, no detailed breakdown by specific ethnic groups nor geographies smaller than regions was provided.

The ONS's (2020b) report on homeworking also mentioned that those in professional and managerial occupations requiring higher qualifications and experience were more likely to work from home. The ONS, based on the U.S. data, Annual Population Survey and Annual Survey of Hours and Earnings (pre-pandemic) assessed that certain occupations, especially with higher hourly wages, were more likely to be able to work from home (ONS, 2020g). The factors that mostly contributed to not being able to work from home included specific location of the job, the amount of face-to-face interaction, requirements of physical activity and use of tools and protective equipment (*idem*.).

### 2.2. Effects on care workers and parents

As described in Sections 1.3 and 2, a disproportionate number of ethnic minority workers were frontline care workers and exposed to health risks related to COVID-19. Based on the LFS data, Cominetti et al. (2020) assessed that around half of frontline carers were paid less than the real Living Wage,^15^ which, in turn, affected the ethnic minorities in those occupations.

Cheng et al. (2021) drew attention to the stress faced by working parents who, because of the school closures, had to spend additional time on childcare and home schooling. Cheng et al.'s (2021) analysis of the COVID-19 Understanding Society data showed that this burden was not equal across households of differing income and between men and women. In particular, working mothers were found to be experiencing worse financial and mental health outcomes. Further, Lee (2020) highlighted the lack of specific economic provision for families in the policy response to COVID-19, finding that over two-thirds of parents reported having their work affected by COVID-19, compared to around a third of non-parents. That Bangladeshi and Pakistani minorities were more likely to have dependent children than those of other ethnicities (Platt and Warwick, 2020) is therefore of note, particularly given that working-age adults with dependants primarily faced employment and financial vulnerabilities (Mikolai et al., 2020). These difficulties were further compounded by the fact that individuals of ethnic minority background were less likely to have a partner in paid work whose income could buffer against a period of financial hardship (Platt and Warwick, 2020).

### 2.3. Food insecurity

Manchester's food banks saw unprecedented numbers of people in need of support, with Manchester South Central Food bank, which supports southern neighborhoods of Hulme, Whalley Range, Old Trafford and Moss Side, reporting a 250% increase in demand in August 2020, compared to the previous year (Maidment, 2020). Similar findings were reported by Graven et al. (2020) for the Bradford District. The authors further explained that some services were unable to cater to various dietary requirements and cultural and religious needs (such as halal food) in multi-ethnic and multi-faith areas. They also recommended that barriers to inclusion related to ethnicity and religion require better understanding and addressing by policymakers.

In April 2020, Loopstra (2020), through a survey fielded for the Food Foundation (sample size around 4,000), identified that the pandemic had worsened food insecurity in the UK in three ways. Firstly, it exacerbated the situation of those already economically vulnerable; secondly, it created new vulnerability due to income losses; and finally, it created new dimensions of food insecurity when people were unable to acquire food due to self-isolation and shielding. The authors also found that the Black and Asian population were at a significantly higher risk of food insecurity than those of White or Mixed ethnic background, especially arising from economic hardship.

Barker and Russell (2020) discussed issues in the government's response to the food security crisis in Britain, including provisions for children who qualified for free school meals, and emergency food aid for older people with pre-existing health conditions. Both groups were provided food parcels that received public criticism for their nutritional content. In particular, for the older adults, many had comorbidities of diabetes, obesity and cardiovascular disease, with the food provided likely to exacerbate these issues. The authors also cited a heavy reliance on the voluntary sector to feed the food-insecure as a primary cause of the financial crisis in the charitable sector, compounded by reduced donations. The government's response to children's food insecurity was limited to summer holidays 2020 (following the extensive campaigning by footballer Marcus Rashford, Sustain and Good Law Project) and further support for free school meals finished in autumn 2020 (for a detailed description see McIntyre et al., 2022). However, provisioning of free school meals, especially outside of the term-time, was limited. For instance, Parnham et al. (2020) used a sample of 635 children from the COVID-19 Understanding Society. They found that about half of these children, all eligible for free school meals, did not have access to the scheme during the April 2020 lockdown.

Power et al. (2020) highlighted the 5-week wait for the Universal Credit benefit as a key factor in increases in food insecurity during the pandemic. The authors also drew light to the effect of austerity policies prior to the onset of the pandemic, illustrating that this increased food insecurity is occurring against a backdrop of growing inequality in access to food. Whilst the authors noted the impressive response of the government to the economic crisis brought by COVID-19, they argued that this underscored that it is the government's responsibility to protect population health and household incomes.

### 2.4. Homelessness and housing insecurity

Lombard's (2021) qualitative study of low-income economic migrants' experiences of housing precarity in Manchester gave a voice to the subjects of such reports. Many interviewees reported tolerating poor housing conditions and overcrowding as a means to attain future housing objectives such as saving for a deposit. This type of inequity is crucial to understanding the disproportionate impact of the pandemic on ethnic minorities. 11% of non-White British households in the North West were overcrowded over the period April 2016–March 2019, in comparison to 1% of White households, with the rate of overcrowding for Bangladeshi, Pakistani, and Black African households at 24, 18, and 16%, respectively (ONS, 2020a). Such numbers demonstrated these groups were disproportionately exposed to the risk of contracting the virus from a member of their household due to the unfeasibility of social distancing within the home.

The UK government requested local authorities to assist the homeless. “Everyone in” was a scheme that initially sought to provide emergency accommodation to the homeless and rough sleeping, facilitating their self-isolation and preventing a wider spread of COVID-19 during the first national lockdown in March-June 2020 (Cromarty, 2021). The scheme was credited with preventing around 21,000 infections and 266 deaths amongst homeless people in the first wave of the pandemic (March-May 2020) but it was less effective in the second wave in winter 2020/21 (Lewer et al., 2020; Doran and Tinson, 2021). According to the Mayor of Greater Manchester Combined Authority, Andy Burnham, at least 1,500 placements were created under the first phase of the “Everyone in” policy (Burnham, 2020). Greater Manchester local authorities also adapted the already existing initiative, “A Bed Every Night,” that supplied more long-term accommodation and created additional bed-spaces (Garvie et al., 2021).

Crankshaw et al. (2020) praised the rapid response of the UK government to provide additional funding to get rough sleepers off the streets. However, the authors urged the need to focus on the sustainability of the support for the homeless and those in temporary accommodation. The summary drew particular attention to those in emergency accommodation with No Recourse to Public Funds (NRPF) due to their immigration status, who might have lost work throughout the pandemic and who did not qualify for the Coronavirus Job Retention or Self-employment Income Support Schemes. There have been subsequent calls to remove the NRPF restrictions for the hundreds of such migrants in Greater Manchester's localities (Greater Manchester Immigration Aid Unit, 2020). This sentiment has been backed by leaders including Manchester's Mayor Andy Burnham in an open letter to the Home Office in December 2020, which urged the government to review the No Recourse policy so that it can protect people from rough sleeping (GMCA, 2020b). Furthermore, asylum seekers relied heavily on services provided by charities (All Party Parliamentary Group for Refugees, 2017). Given reduced access to such services, with many moved online over the course of the pandemic, digital exclusion from such services constitutes another cause for concern.

Based on a survey during the first lockdown conducted in Bradford, a multi-ethnic and deprived area of northern England, Dickerson et al. (2020) found that many families from ethnic minorities lived in poor quality or overcrowded housing. Insecure food and employment were also a common occurrence amongst those surveyed, particularly for the self-employed, furloughed, and unemployed. The lessons from the survey are likely to apply to the deprived areas of Manchester with similar population distribution regarding occupations, ethnicity and level of deprivation.

### 2.5. Personal finances and financial security

Brewer and Tasseva (2020) analyzed the UK government's early policy response to COVID-19, estimating its impact on household incomes in April and May 2020 using microsimulation methods. They projected average income losses of 6.9% for the UK households. They also posited that changes to Universal Credit, Job Seekers Allowance and the tax system could reduce the shock of the economic consequences of the pandemic.

Bhattacharjee and Lisauskaite (2020) also used a microsimulation to project the number of households in destitution in the UK, predicting that destitution was three times higher in the second quarter of 2020 than the non-COVID counterfactual level. In contrast, forecasts by Bronka et al. (2020) for June 2020, based on the 2016 Input-Output and LFS data, suggested that the intervention of the government would contain the reduction in average household disposable income from −3% to −1%. Their predictions also pointed to a reduction in poverty of more than 1 p.p. relative to pre-COVID levels, due to increased Universal Credit, despite predictions of a reduction in employment of around 25% and a reduction in yearly GDP of around 2%. However, these estimates were based on an 8-month timeline, where the duration of the income shock was expected to be 2 months of strict lockdown at 100% of the estimated effect, 2 months of partial lockdown at 50% of the estimated effect and a further four months in a recovery phase at 25% of the estimated effect. This did not reflect the reality of the pandemic with subsequent lockdowns in November 2020 and January-March 2021.

Gathergood et al. (2021) conducted a geographical analysis of consumer spending in the UK in the second half of 2020. The authors utilized Fable Data,^16^ a real-time source of consumption data that contain postcode-level geo-tags for cardholder and store addresses. The authors found evidence of regional unbalance, with the strongest recovery in spending seen in the commuter belt areas in outer London and surrounding localities.

The social stratification of the impact of the pandemic and lockdown was highlighted in a study conducted by Mikolai et al. (2020). The study illustrated the presence of a multi-dimensional index of inequality that often intersected within households, particularly in those of working age. The study evidenced regional variation in household-level vulnerabilities, indicating the need for a nuanced approach to mitigating the effects of the pandemic; however, they stress the need for further spatial disaggregation. The analysis by Witteveen (2020; see Section 2.1.2) also found that lower earning groups were over twice as likely to experience economic hardship when compared with the highest quintile earners.

Mogaji (2020) identified early in the pandemic that financial vulnerability would be a key issue for countries and individuals. The study highlighted the important role of the government as well as banks in supporting these individuals, drawing attention to the potentially harmful actions that can result from financial vulnerability, including gambling and the use of payday loans. Indeed, a report from the Financial Conduct Authority (2021) found that between March and October 2020, the number of adults with characteristics of financial vulnerability increased by 15% compared to the February figure. In February 2020, these adults were more than twice as likely to utilize high-cost credit options. Further, 38% of adults saw their financial situation worsen overall due to COVID-19, with particularly strong impacts on the self-employed, adults with a household income of less than £15,000 per year, and BAME adults.

Benzeval et al. (2020) congruently utilized the COVID-19 Understanding Society study (ISER, 2021) to estimate the prevalence of mitigation strategies for the financial strain of the pandemic. These opportunities were most limited for those most in need. The study found that individuals classified as of BAME background were more than twice as likely to have resorted to borrowing than the non-BAME population. Further, BAME individuals who lost hours due to the pandemic were 14 p.p. less likely to be furloughed and 13 p.p. more likely to be unemployed than the non-BAME individuals, largely agreeing with findings of Crossley et al. (2021; Section 2.1.2).

Hu (2020) also used the COVID-19 Understanding Society survey to examine inequalities in economic wellbeing amongst natives and migrants classified by their ethnicity. Results indicated that BAME migrants in the UK were more likely to have experienced job loss, whilst BAME natives were less likely to enjoy employment protection such as furloughing, compared to the UK-born White British respondents. The UK-born White British respondents were also less likely to experience income loss and face increased financial hardship during the pandemic than BAME migrants. While the study relied on a relatively small sample size, it highlighted a need to consider the migrant status in discussions and interventions to mitigate the economic impact of the pandemic on ethnic minorities. This is especially relevant for Greater Manchester being home to over half of the foreign-born residents in the North West (The Migration Observatory, 2014), and considering the COVID-19-induced challenges unique to migrants such as those with No Recourse to Public Funds (Section 2.6).

### 2.6. Access to benefits and Universal Credit

Both Harris et al. (2020) and Machin (2021) explored the impact of additional funding, which was announced for the UK social security system and included significant amendments to Universal Credit, disability, carers and sickness benefits. Millar (2020) and Machin (2021) argued that the response did not go far enough and that one of the most significant weaknesses in these measures was the failure to rectify the 5-week delay for the first payment of Universal Credit. Mackley (2021) indicated that in month ending 9 April 2020, the Universal Credit claims increased by a million more people than the standard monthly volume. Due to this sharp rise, people faced problems in identifying themselves through the system, given the implemented policies seeking social distancing. To reduce the frequency of UC claims and to facilitate UC claims, the identification of people started being done by telephone, and the support rate was increased. As mentioned in Section 2, the conditions to request a Universal Credit were temporarily suspended.

Emerson et al. (2021) investigated the economic impacts of the lockdown using the COVID-19 Understanding Society (ISER, 2021) data, focussing their inquiry on employment and financial security for those with disabilities. Results indicated that working-age adults with a disability were more likely to be working reduced hours and experiencing higher levels of financial stress when compared to those without a disability. The Women's Budget Group (2020) found that of those who were out of work due to disability or were retired, over twice as many BAME women and men (42.5% and 48.3%) reported that they had recently lost support from the government than White women and men (12.7% and 20.6%). It is thus crucial that intersections of ethnicity and disability are considered in policy response.

**Figure 2:**
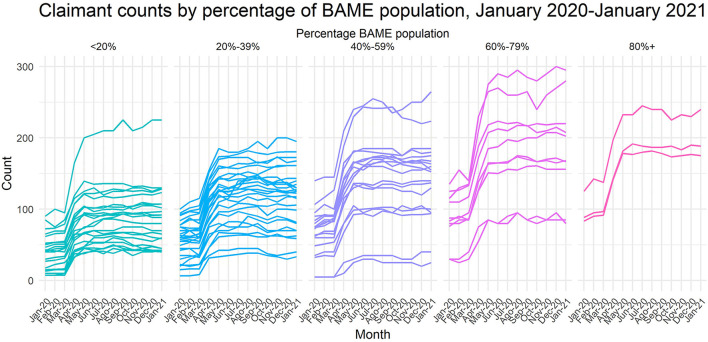
Claimant counts by percentage of ethnic minority (BAME) population in a given LSOA, Manchester, 2011 Census (available when this analysis was originally carried out). Source: NOMIS Claimant Count data. There is a significant association between the number of claimants in LSOAs and the percentage of ethnic minority population in each of them, that is, an LSOA with a higher ethnic minority population is more likely to have more claimants (χ^2^ = 40,393,080, df = 1,124, *p* < 0.001). The BAME population is defined here as a sum of population of Mixed/multiple ethnic groups, Asian/Asian British, Black/African/Caribbean/Black British, and Other ethnic groups; all White population groups are excluded.

As shown in Table 1, Greater Manchester saw vast increases in claimants for Universal Credit across all ten boroughs, with a surge of 90% between March and July 2020. The largest increase in percentage points (as a proportion of residents aged 16–64) was noted for Oldham and Manchester, by 4.3 (to 9.4%) and 4.2 (to 8.7%) percent point increases over these 4 months. Meanwhile, in the UK, an increase was from 3.0 to 6.3% and in Greater Manchester from 4.3 to 7.8%. Further, Manchester, Oldham and Rochdale local authorities all had around 10% of the working age population claiming unemployment benefits, whilst Greater Manchester's average was 8%, much higher than the 2020 national equivalent of 6.4% (GMCA, 2020a). These changes are illustrated in Figure 2, which presents the counts of benefits claimants between January 2020 and 2021 in LSOAs grouped by the percentage of BAME^17^ population residing in them are presented. We observe that the highest increases of the claimant counts were recorded in the LSOAs with 40–80% of the BAME population. There is also a positive association between the percentage of BAME in the LSOA and the count of benefit claimants.

Further analysis of Universal Credit claimant count data in Manchester local authority between May and December 2020 revealed that four out of five LSOAs with the largest increases have majority BAME populations (see Supplementary Figures 2, 3). This is true for both in- and out-of-work claimants, suggesting that employment does not guarantee financial stability for these groups (Nomis, 2021). Qureshi et al. (2020), following Sandhu (2016), brought to light the intrinsic racial inequities in access to Universal Credit, including the two-child limit and barriers to application such as digital exclusion, further exacerbated by language barriers. Further protection is thus necessary for ethnic minority women who were unduly affected by such constraints.

Supplementary Figures 5–8 illustrate borough-wise the prevalence of each minority ethnic group, providing a detailed background account to support careful consideration of reasons behind the higher claimant count rise in those areas with more people from ethnic minorities. Policies that aim to mitigate ethnic effects of any kind need to respond sensitively to the detail of the group sizes in each borough, as well as different mechanisms that may operate on and via these groups in accessing health care, responding to vaccination schemes, taking up social security and engaging with authorities.

## 3. Discussion and lessons learnt

The onset of the COVID-19 pandemic and subsequent measures taken to mitigate its spread occurred against a backdrop of inequity. As a result, ethnic minorities have faced worse labor market outcomes due to the pandemic and restrictions than White people across the UK. The government's provisions have not included enough consideration for prior inequities in the labor market and the ways in which ethnic minority groups were uniquely impacted by the pandemic because of these. These findings are universally relevant at a time of a crisis, such as the cost of living crisis since late 2021 (Marmot, 2022).

Differentiation within the minority ethnic groups is likely to be a source of confusion and inaccuracy via two interpretative difficulties. First, the prevalence of the groups is highly clustered at local authority and lower geographies level in the UK, so policy initiatives set up at a national level might be spread too thin. Secondly, the class and gender differentiation within each minority group within a borough like Manchester city would mean some members of the group are not experiencing the same health and social-security situations as the majority of them. The situation in Greater Manchester can be considered either hyper-diversity or intersectional diversity. The intersection is acute when economic class polarity is high and rising, as it currently is in the UK cities (Lee et al., 2016). The gender differences in turn are culturally-influenced with each ethnic group sharing some cultural similarities. As a result close study of the groups named in Supplementary Figures 5–8 is valuable before targeting and refining policy actions.

A recognition of the intersecting and multiple vulnerabilities faced by ethnic minority individuals in both Manchester City and Greater Manchester area is integral to producing an effective policy response. As Platt and Warwick (2020; p. 286) state, “There is no single narrative that can describe or account for the impacts of the current crisis on all minority groups.” Pre-pandemic, these groups were already disadvantaged in numerous ways, facing lower levels of employment, and when employed more likely to work in insecure, low paid and undervalued jobs.

Nazroo et al. (2020) reached similar conclusions in their review for Greater London Authority. They indeed found substantial COVID-19-related inequalities, in terms of risk of infection, complications and mortality, as well as the negative economic, social and psychological consequences of Government policies to mitigate the health impacts of the pandemic. These inequalities specifically related to ethnic minorities, people with disabilities, and women. While women had lower risk of COVID-19 mortality, they experienced disproportionate economic, social and psychological impacts of the pandemic. The authors stressed that it was the well-documented, pre-pandemic social and health inequalities that only deteriorated during the pandemic for those in most precarious situation.

Throughout this review, particularly negative labor market impacts have been noted for those of Bangladeshi and Pakistani ethnicity due to them being represented in high numbers in self-employment and/or in shutdown sectors, in conjunction with a higher prevalence of single-earner households, low levels of female employment, (Section 2.1.2), and higher likelihood of having dependent children (Section 2.4). However, detailed analyses of the outcomes for specific ethnic minorities in areas smaller than a local authority were exacerbated, if not virtually impossible, due to the lack of detailed data on economic and health outcomes of those populations. The data on ethnic composition relies on censuses, supplemented with LFS/APS data (Supplementary material section 1). However, the relatively small sample sizes, releases with around half-year delay, as well as difficulties in fielding surveys during lockdowns, made these data unreliable for designing timely and effective policy responses.

The other popular data source on outcomes related to the COVID-19 impacts was Understanding Society UK Household Longitudinal Study (UK HLS) and its COVID-19 modules (see Supplementary material section 1). While these data permitted analyses for ethnic minorities at the LSOA level in principle, in practice the sample sizes per LSOA were very small and did not allow meaningful inference and comparisons within, e.g., city of Manchester. Further difficulties arose because the UK HLS survey did not aim to accurately represent each borough of the Greater Manchester conurbation, and may have missed out some neighborhoods through having selected clusters of areas for entering the survey. Thus, conclusions about the COVID-19 impacts on ethnic minorities are typically based on aggregated data. Another important source of data on employment and other economic impacts is Labor Force Survey. While it has larger sample sizes in smaller areas, the resulting estimates have been still characterized by relatively wide confidence intervals, even for local authorities (Section 2.1). Both of these issues were further exacerbated by the change of the interviewing model from face-to-face to telephone (Supplementary material section 1).

A potential source of information on economic outcomes was the data on Universal Credit (Section 2.3). These data are available at a relatively low geography level (LSOA) and at a monthly interval. Further disaggregations (by age and sex) are less reliable due to rounding. These data could potentially have been used to monitor the increases in claimant counts and claimant rates in the city of Manchester and other areas smaller than a local authority (such as LSOA). We showed that large increases were noted in areas of Manchester with very high proportions of ethnic minorities. Another useful source of information for smaller areas are targeted surveys, such as those carried out in Bradford (Dickerson et al., 2020; Graven et al., 2020).

These data, however, would not be sufficient to systematically analyse the impact of the pandemic and other crises on the particularly vulnerable migrant populations and those with No Recourse to Public Funds.

In the UK and other countries, a post-pandemic 2021 round Census is being released, including a range of Census products which can respond to the ethnicity issues and intersectionality that emerged in this review. In tabular products, the provider can revise the categories used for summary tables, aiming for multidimensionality. However, at the time of the COVID-19 outbreak it was the 2011 Census data that were most recent. As we show in Section 1, Manchester's ethnic composition changed considerably since 2011, thus, these data may have not been adequate to assess the impacts of the pandemic for specific ethnic minorities. In random-sample surveys, easy access is offered in the UK to users via secure data outlets, so that geographic granularity is accessible at a low level whilst the outputs are monitored to ensure privacy and anonymity of respondents. Here, the ethnicity and social-security uptake are classed as sensitive personal data and there is an expansion of rules and procedures (plus opportunities) to use these data for creating informative outputs rather than the data with local-level identifiers being off-limits. Users of secure data may be part of a future solution to measuring and analyzing the long-term effects of the pandemic including at low-level geographies.

Statistical models aiming to explain outcomes, keeping ethnicity and cultural or national origin in mind along with current location, have expanded in recent years to allow a mixed individualist and ecological analysis of data (e.g., Olsen et al., 2020). Spatial analysis is used to examine ecological correlations such as the uptake of benefits by locality, illustrated here. Current developments embed this line of argument into wider statistical models, taking up mediation or interaction effects to develop sophisticated lines of argument. Such research will translate into impact on local practitioners once it is possible to communicate findings to those who are able to change the situation facing socially-excluded groups and those facing multiple marginalization. The Census itself can be used in combination with other surveys using new data-combining methods, too.

Possible alternative approaches used here included relying upon qualitative methods and ad-hoc surveys (Section 2.6). In general, the scarcity of data by ethnicity/migrant status presents a significant gap that should be addressed in order to effectively mitigate the economic fallout of COVID-19 for those who most need it. The value of new data sources that accurately cover minority ethnic groups should cut across not only health but also well-being, welfare and access to a range of civic rights.

## Author contributions

AW and WO contributed to the conception of the review. AW prepared a final manuscript. RA performed the review and wrote the first draft of the manuscript. AA-C performed a review of data sets and statistical analyses. AA-C and MI wrote sections of the manuscript. All authors contributed to the manuscript revision, read, and approved the submitted version.

## Footnotes

^1^Middle Layer Super Output Areas (MSOA) and Lower Layer Super Output Areas (LSOA) are a geographic hierarchy of areas with approximately consistent sizes of populations. For MSOAs, the mean population is 7,200, whereas for LSOAs it is 1,500.

^2^https://www.ethnicity-facts-figures.service.gov.uk/style-guide/writing-about-ethnicity; see also 2021 Census ethnicity classification at https://www.ons.gov.uk/census/censustransformationprogramme/consultations/the2021censusinitialviewoncontentforenglandandwales/the2021censusethnicgroupstakeholderfollowupsurvey/furtherinformationthe2021censusethnicgroupstakeholderfollowupsurvey.

^3^ https://civilservice.blog.gov.uk/2022/05/19/why-weve-stopped-using-the-term-bame-in-government/

^4^Local Authority (LA), also referred to as a council or borough, is a local government in England. There are 333 LAs in England. Details: https://www.gov.uk/guidance/local-government-structure-and-elections.

^5^ https://www.nomisweb.co.uk/

^6^The Living Wage is calculated by the Resolution Foundation and is based on evidence about living standards in the UK. In 2015 this was £7.85 per hour.

^7^ https://assets.publishing.service.gov.uk/government/uploads/system/uploads/attachment_data/file/966138/Levelling_Up_prospectus.pdf

^8^Introduced by the Conservative Party following the 2007-8 financial crisis, austerity measures affected numerous facets of public spending, including a reduction in spending on welfare via the Welfare Reform Act 2012 and reductions in local government funding.

^9^ https://www.gov.uk/government/publications/city-deal-greater-manchester

^10^The first wave was defined between 24/01/2020-−11/09/2020; the second wave 12/09/2020-−31/03/2021 (ONS, 2020e).

^11^The Townsend score measures deprivation with unemployment, non-car ownership, non-home ownership and household overcrowding (Townsend and Davidson, 1982).

^12^Health Protection Regulations 2020; also https://www.gov.uk/government/publications/covid-19-stay-at-home-guidance/stay-at-home-guidance-for-households-with-possible-coronavirus-covid-19-infection (accessed 15/04/2021). The regulation applied in England and Wales but not in Scotland nor Northern Ireland.

^13^Note, that these data were not available until 28 June 2022. UK's 2021 Census provides detailed information on industry and socio-economic status of population in small areas, https://www.ons.gov.uk/employmentandlabourmarket/peopleinwork/employmentandemployeetypes/bulletins/industryandoccupationenglandandwales/census2021. However, these data were not available when the pandemic broke out. Another source of individual-level information on occupations and industry is Business Register and Employment Survey but these data are only available for the place of employment, not residence, and hence may not be adequate to compare with areas of residency as listed in the Census.

^14^https://www.ethnicity-facts-figures.service.gov.uk/work-pay-and-benefits/employment/self-employment/latest (accessed 29/05/21).

^15^At the time of Cominetti et al.'s (2020) study this was £10.75 in London and £9.30 across the rest of the UK.

^16^More information about these data is available at https://www.fabledata.com/.

^17^The BAME population are defined here as a sum of population of Mixed/multiple ethnic groups, Asian/Asian British, Black/African/Caribbean/Black British, and Other ethnic groups; all White population groups are excluded. The data are from 2011 Census that were available at the time of the analysis.
