# Supplementary material for: The economic impact of the COVID-19 pandemic on ethnic minorities in Manchester: lessons from the early stage of the pandemic
Source: Front Sociol. 2023 May 19;8:1139258. doi: 10.3389/fsoc.2023.1139258 (PMC10237338; doi:10.3389/fsoc.2023.1139258)
Supplement: Supplementary file 1 [file Data_Sheet_1.docx]

Supplementary Material

# Data Sources

This section describes the datasets that are currently available and most relevant to research on this topic. They constitute economic data that can be broken down into ethnic groups and enable data analysis mostly at a Local Authority level. Some of these datasets contain questions directly related to COVID-19. Others do not have specific questions regarding COVID-19 but their application in the period before the pandemic and during it allows the analysis of the differences between both points in time. The data sources are summarised in Table A.1.

The **Annual Population Survey** (APS) is an annual survey with the largest sample in the UK (320,000 individuals and 150,000 households) ^[[1]](#footnote-2)^. This survey covers topics on individual demographics including ethnicity, labour market status, and work characteristics (such as employment by occupation, sector, qualification level, self-employment, unemployment by qualification level, length of time spent in unemployment, economic inactivity and average hourly pay). This survey has specific person and household datasets that enable analysis at a local authority level. However, the sample sizes render the confidence intervals relatively wide for meaningful analyses of, for example, unemployment rates, over time at small geographies. APS uses the ethnic categories from the 2011 Census with some modifications when displaying results. This survey aggregates Black, Mixed and Other ethnic groups into single group. It also groups Indian, Pakistani and Bangladeshi in a unique category, separate from other Asian ethnic groups. By contrast, it shows results separately for the White British and Other White. The most recent data are from September 2020, with the next release scheduled for 20 April 2021.

The **Labour Force Survey** (LFS) has the same core questions as the APS. However, the LFS sample size is smaller than the APS sample (95,000 persons and 39,000 households). Despite this, the LFS also allows analysis at a local level (LA). The LFS provides annual information on economic activity, occupation, qualification and hours worked. LFS information can be broken down by – amongst other individual characteristics – ethnicity. There are LFS weekly and monthly estimates, but they are not at a local level. During COVID-19 pandemic, the LFS was affected by the change of the mode by moving from face-to-face to telephone interviewing. This affected the response and non-responses rates and biases and required adjustments to the weighting procedures, possibly affecting final estimates.

Since March 2020, the ONS has implemented the **Labour Market Survey** (LMS), for which samples are drawn from the Postcode Address File. This survey is applied quarterly and aims to test a mixed-mode design for a household survey^[[2]](#footnote-3)^^[[3]](#footnote-4)^. Based on the LMS, the ONS released a report Coronavirus and Homeworking in the UK, which looks at homeworking patterns in the UK disaggregated by sex, age, region and ethnicity.

**Understanding Society: the UK Household Longitudinal Study** (UKHLS) is a large national probability-based household survey that has been collecting and publishing data annually since 2009. Since April 2020, this study included questions regarding the impact of the pandemic on participants’ welfare, in domains such as employment, finances, financial security and coronavirus illness. Six waves have been collected during 2020 (in April, May, June, July, September and November). Data is available at a LSOA level (within secure access) and can be disaggregated by ethnicity. Amongst the statistics that the UKHLS enables to produce is the **Income Dynamics Statistics** reported by the Department for Work and Pensions (DWP). This specific dataset is about persistent low income and changes in income of individuals and households. These data can be disaggregated by local authority level and ethnicity.

The **Opinions and Lifestyle Survey** (OPN) measures the impact of COVID-19 on people’s work for around 4,000–4,500 individuals in Great Britain^[[4]](#footnote-5)^. The survey also includes questions related to taxation, food safety, and health. The survey has been collected weekly since 20 March 2020 and its last release was on 20 March 2021. Data can be obtained by local authorities and ethnicity.

The **Family Resources Survey** is an annual survey, with results published by the Department for Work and Pensions. This survey includes economic variables (e.g. household income, state support, and sources of household income) broken down by ethnicity. Some of the variables can be found by local authority level (for example, State support). No questions specific to COVID-19 were included in the latest release (25 March 2021). This last release corresponds to the financial year April 2019 to April 2020.

The **Business Insights and Conditions Survey** (BICS) provides information on financial performance, workforce, prices, trade, and business resilience. This survey has been collected fortnightly for 26 waves. Since the seventh wave, the methodology of this survey has changed. Data from wave 7 (2 July 2020) to wave 26 (25 March 2021) are now available^[[5]](#footnote-6)^. Experimental weighted regional and local estimates were calculated from the BICS. Based on these estimates, the ONS released a report on the Business insights and Impact on the Subnational UK Economy from 21 September 2020 to 10 January 2021^[[6]](#footnote-7)^. In addition, unweighted estimates were obtained from BICS, conforming the Business Impact of COVID-19 Survey (BICS) results. These results were produced fortnightly from 9 March 2020 to 1 November 2020. This COVID-19-specific survey was discontinued^[[7]](#footnote-8)^. Given that the unit of analysis in BICS is businesses, this dataset does not account for ethnicity.

The **Annual Survey of Hours and Earnings** (ASHE) is an annual survey that collects information on weekly pay, basic pay, overtime pay, gross hourly pay, gross annual pay, annual incentive, total paid hours, and basic paid hours. Since 21 April 2021, this survey was modified to account for the Coronavirus Job Retention Scheme (“furloughing”). The modifications included asking whether the employee was furloughed fully or partly and asking employers for payments due to furloughs. These data allow the data analysis at a local authority level, but it only disaggregates information by sex.

Additionally, some data produced by the Department for Work and Pensions (DWP) enable the analysis of the economic impacts of COVID on the BAME population at a local level. One of the most important datasets is the **Claimant Count and Flow** data. These data provide monthly information on the number of people claiming Jobseeker’s Allowance (JSA) and those claiming Universal Credit (UC) (such as those claiming benefits due to being unemployed)^[[8]](#footnote-9)^. The DWP collects these data by individual characteristics (e.g. age, duration, ethnicity, occupation) and has georeferenced data by using postcode or output areas. The last aspect enables the analysis at a local level.

The **English Longitudinal Study of Ageing** (ELSA) collects information on individuals aged over 50 years old. Due to the current pandemic, ELSA has collected data on the effects of COVID on older people, focusing on the financial consequences of the coronavirus pandemic.

# Universal Credit claimants – a case study

In Supplementary Figure 1, we observe that the association between the percentage of the BAME population within the LSOAs and the number of Universal Credit claimants, for both employed and unemployed. The wards with the highest number of claimants are consistent in both groups and include Moss Side, Longsight, Levenshulme, Harpurhey, Gorton & Abbey Hey, Clayton & Openshaw, and Miles Platting & Newton Heath. The same wards have seen the highest rates of benefits claimants (Supplementary Figure 2). The wards with the lowest counts of claimants include Deansgate, Didsbury West and East, Chorlton, Piccadilly and Withington. Supplementary Figure 3 presents maps of the percentage of BAME population in LSOAs (Panel a), total counts of benefits claimants (b), counts of Universal Credit claimants in employment (c), and counts of Universal Credit claimants unemployed (d), respectively. We observe that LSOAs with a high proportion of BAME population are in the north-western part of Manchester Local Authority area (Panels a and b).

# Supplementary Figures and Tables

## Supplementary Tables

Supplementary Table 1: A selection of data sources containing information on COVID-19 economic impacts. Note: An updated list of data sources related to COVID-19 are available at the UK Data Service: <https://www.ukdataservice.ac.uk/get-data/themes/covid-19.aspx>; official UK Government data are available at <https://coronavirus.data.gov.uk/>.

| Data | Custodian | Where Available | Variables of Interest | Dates | Link |
| --- | --- | --- | --- | --- | --- |
| Business Impacts of COVID-19 Survey | ONS | ONS | Economic Impacts of COVID | March - November 2020 | [https://www.ons.gov.uk/economy...](https://www.ons.gov.uk/economy/economicoutputandproductivity/output/datasets/businessimpactofcovid19surveybicsresults) |
| Claimant Count Data | NOMIS | NOMIS | Unemployment, Benefits, Geographies | Since 1986 | <https://www.nomisweb.co.uk/sources/cc> |
| Coronavirus and Homeworking in the UK | ONS | UK Data Service | Region, Ethnic Minority Indicators | Apr-20 | [https://www.ons.gov.uk/employmentandlabourmarket/...](https://www.ons.gov.uk/employmentandlabourmarket/peopleinwork/employmentandemployeetypes/bulletins/coronavirusandhomeworkingintheuk/april2020) |
| COVID-19 Vaccinations (Weekly) | NHS | NHS | Age, Dose, Region, Ethnicity, MSOA, Care Homes, NHS workers, Clinically Extremely Vulnerable | Weekly from 11 Jan 2021 | [https://www.england.nhs.uk/statistics/...](https://www.england.nhs.uk/statistics/statistical-work-areas/covid-19-vaccinations/) |
| ELSA COVID-19 Survey | English Longitudinal Study of Ageing | UK Data Service | Financial Situation, Mental / Physical Health, Ethnicity | June/July and Nov/Dec 2020 | [https://beta.ukdataservice.ac.uk/datacatalogue/...](https://beta.ukdataservice.ac.uk/datacatalogue/studies/study?id=8688#!/resource) |
| Family Resources Survey | Department for Work and Pensions | UK Data Service | Income and circumstances of UK households | 2010/11 - 2018/19 | [https://beta.ukdataservice.ac.uk/datacatalogue/...](https://beta.ukdataservice.ac.uk/datacatalogue/studies/#!?Search=Family%20Resources%20Survey&Rows=10&Sort=1&DateFrom=440&DateTo=2021&Page=1) |
| Financial Lives Survey | Financial Conduct Authority | CDRC | Financial Situation, Debt, Home Ownership, Resilience | 2017 & 2020 | <https://data.cdrc.ac.uk/dataset/fca-financial-lives-survey> |
| House Price Index | ONS | ONS | House Prices, Economic effects of COVID |  | [https://www.gov.uk/government/statistical-data-sets/...](https://www.gov.uk/government/statistical-data-sets/uk-house-price-index-data-downloads-december-2020) |
| Mind – The Mental Health Charity COVID 19 Survey | Mind | On request from Mind | Mental Health, Ethnicity, | April/May 2020 | [https://www.mind.org.uk/media-a/5929/...](https://www.mind.org.uk/media-a/5929/the-mental-health-emergency_a4_final.pdf) |
| UK Labour Market Data | ONS | ONS | Labour Market, Ethnicity, Region | Various | <https://www.ons.gov.uk/releases/uklabourmarketjune2020> |
| UKHLS COVID-19 Study | University of Essex | UK Data Service | Financial Situation, Mental / Physical Health, Ethnicity, Local Authority (On Request), Region | April - September 2020 | [https://beta.ukdataservice.ac.uk/...](https://beta.ukdataservice.ac.uk/datacatalogue/studies/study?id=8644) |
| Welfare at a (Social) Distance | University of Kent | UK Data Service | Unemployment, Benefits, Small Geographies | Collection in process | [https://beta.ukdataservice.ac.uk/...](https://beta.ukdataservice.ac.uk/datacatalogue/studies/study?id=8689) |
| OpenSAFELY | University of Oxford | University of Oxford | linked primary care data, COVID-19 outcome, ethnicity, cause of death | Collection in progress | https://opensafely.org/ |

## Supplementary Figures


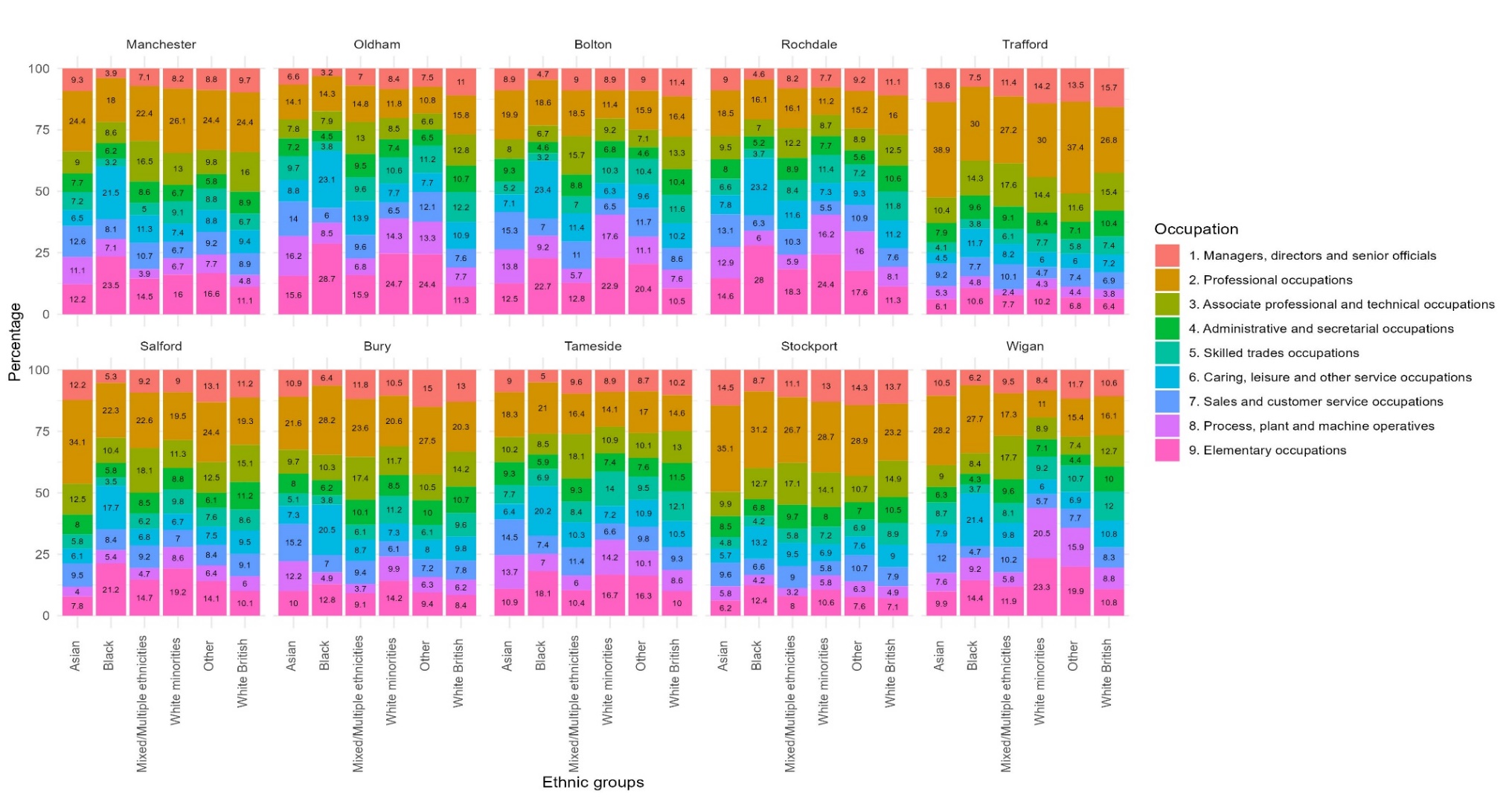


Supplementary Figure 1. Percentage of population by broad ethnic groups and occupation based on the 2021 UK census categories in Greater Manchester boroughs. The **Asian** group is composed of Bangladeshi, Chinese, Indian, Pakistani, and Other Asian. The **Black** ethnic group includes African, Caribbean, Other Black. The **Mixed or Multiple** ethnic groups refer to White and Asian, White and Black African, White and Black Caribbean, Other mixed or multiple ethnicities. **White minorities** are composed of Gypsy or Irish Traveller, Irish, Roma, Other White. **Other** ethnic group category involves Arab and any other ethnicity.

Supplementary Figure 2: Employed and unemployed persons taking Universal Credit, 2020, in LSOAs grouped by ward (rows) and percentage of the BAME population in the LSOA (columns). Source: NOMIS Claimant Count data. There is a significant association between the number of Universal Credit claimants in LSOAs and the percentage of BAME population in each of them, that is, LSOA with higher BAME population are more likely to have more claimants, for both employed (χ^2=2099280, df=1124, p<0.001) and unemployed (χ^2=3985320, df=1124, p<0.001). The BAME population is defined here as a sum of population of Mixed/multiple ethnic groups, Asian/Asian British, Black/African/Caribbean/Black British, and Other ethnic groups; all White population is excluded.


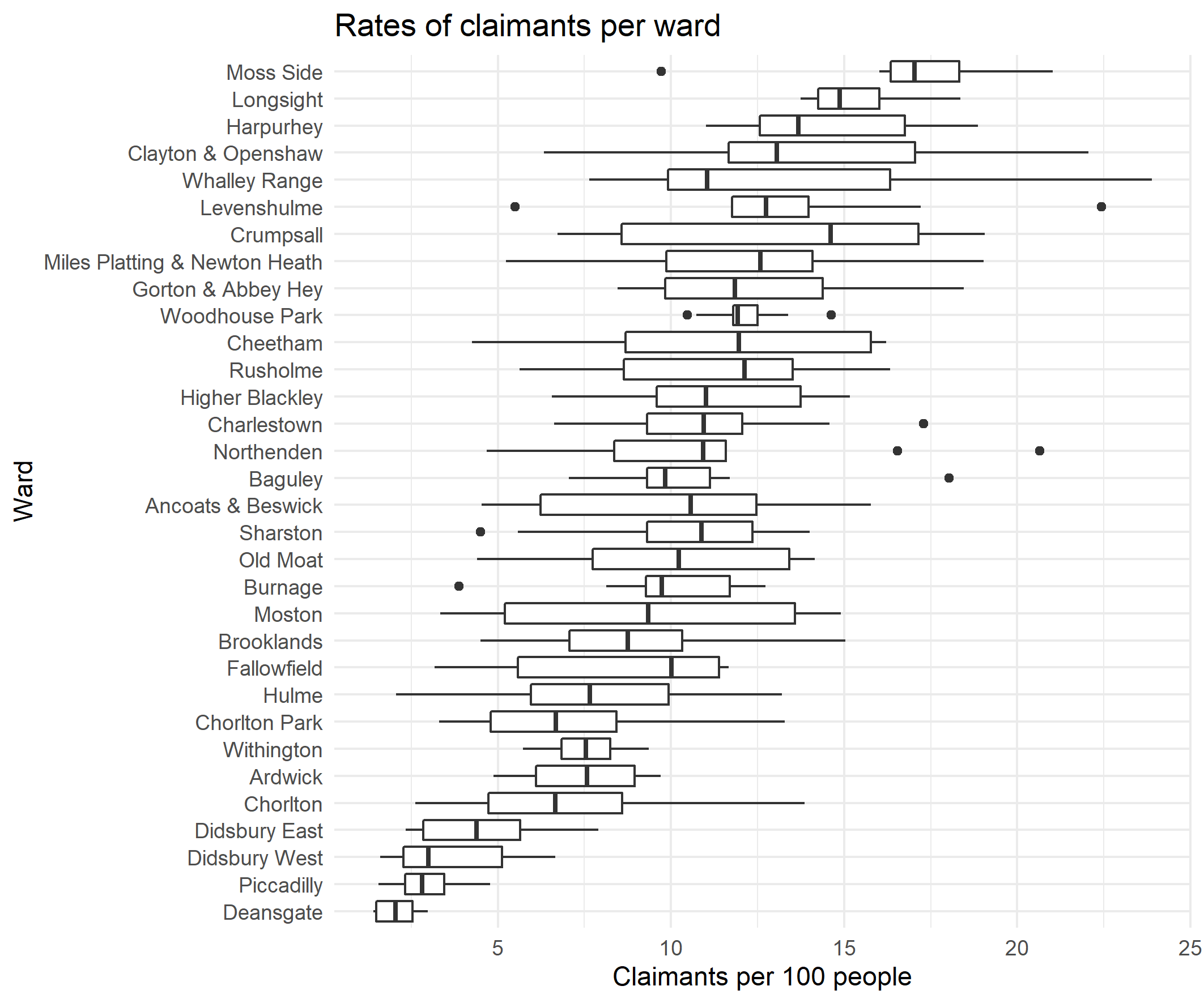


Supplementary Figure 3: Rates of benefits claimants per ward, aggregated over LSOAs, Manchester, January 2020 – January 2021. Source: NOMIS database and ONS Mid-2020 Population Estimates for 2020 Wards and 2021 LAs in England and Wales by Single Year of Age and Sex - Experimental Statistics.

a) b)


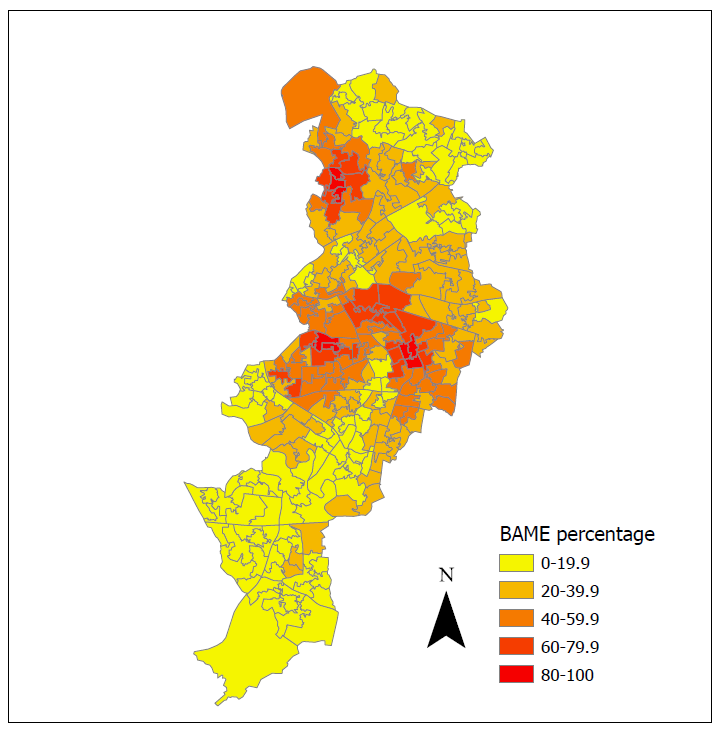

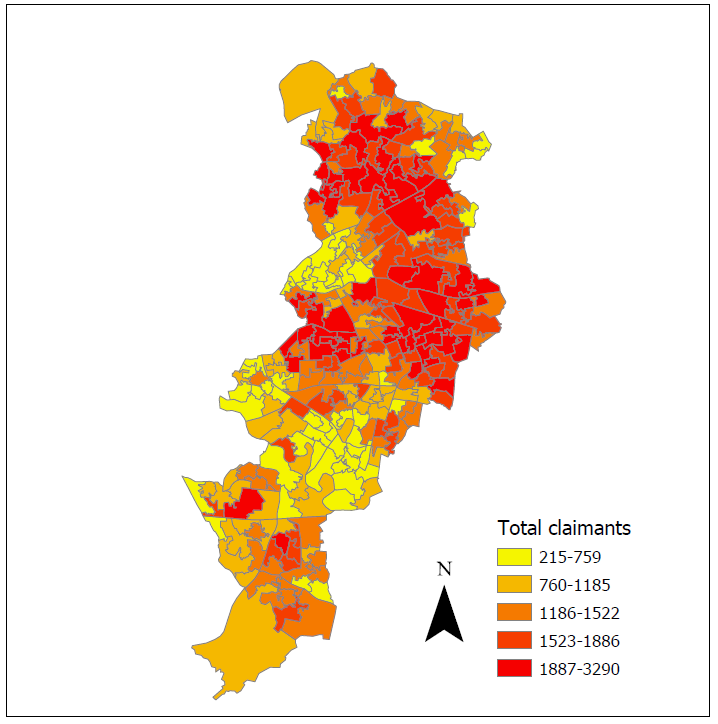


c) d)


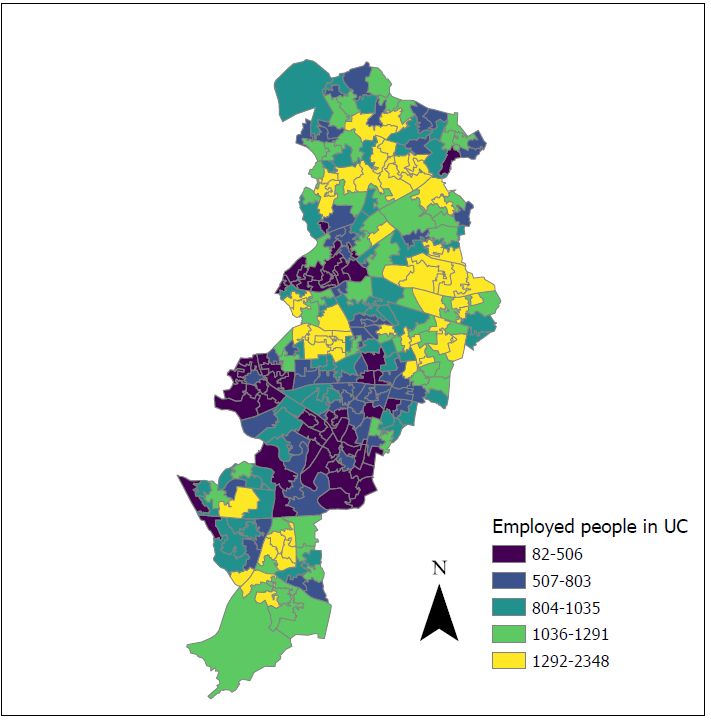

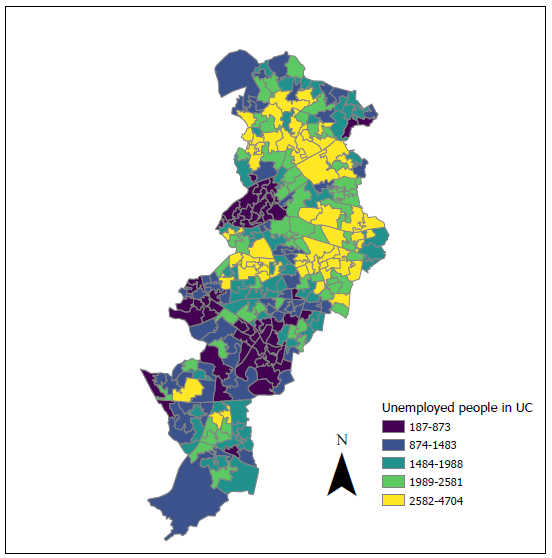


Supplementary Figure 4: Percentage of BAME population in LSOAs (panel a), total counts of benefits claimants (b), counts of Universal Credit claimants in employment (c), and counts of Universal Credit claimants unemployed (d) in Manchester district, 2020. Source: own elaboration using NOMIS database. The BAME population is defined here as a sum of population of Mixed/multiple ethnic groups, Asian/Asian British, Black/African/Caribbean/Black British, and Other ethnic groups; all White population is excluded.


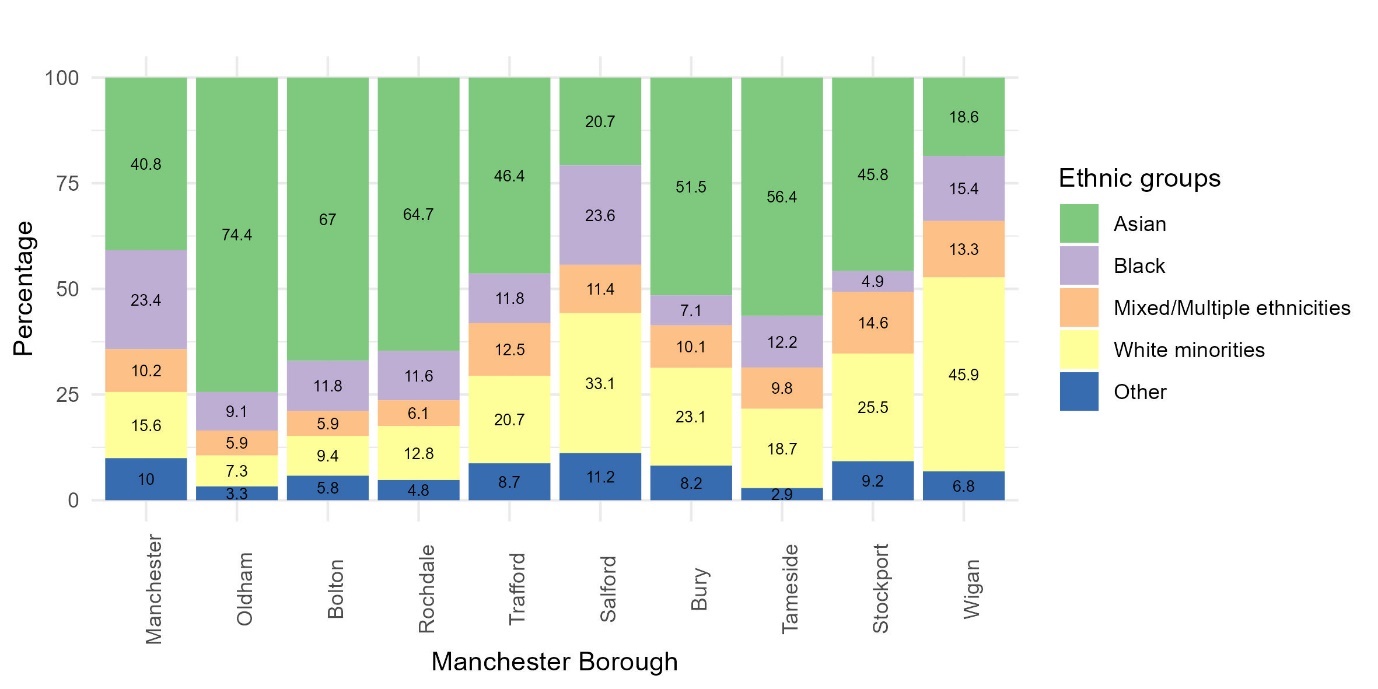


Supplementary Figure 5: Percentage of population by broad minority ethnic groups based on the 2021 UK census categories in Greater Manchester boroughs, excluding the General Population (i.e. White British). The **Asian** group is composed of Bangladeshi, Chinese, Indian, Pakistani, and Other Asian. The **Black** ethnic group includes African, Caribbean, Other Black. The **Mixed or Multiple** ethnic groups refer to White and Asian, White and Black African, White and Black Caribbean, Other mixed or multiple ethnicities. **White minorities** are composed of Gypsy or Irish Traveller, Irish, Roma, Other White. **Other** ethnic group category involves Arab and any other ethnicity.


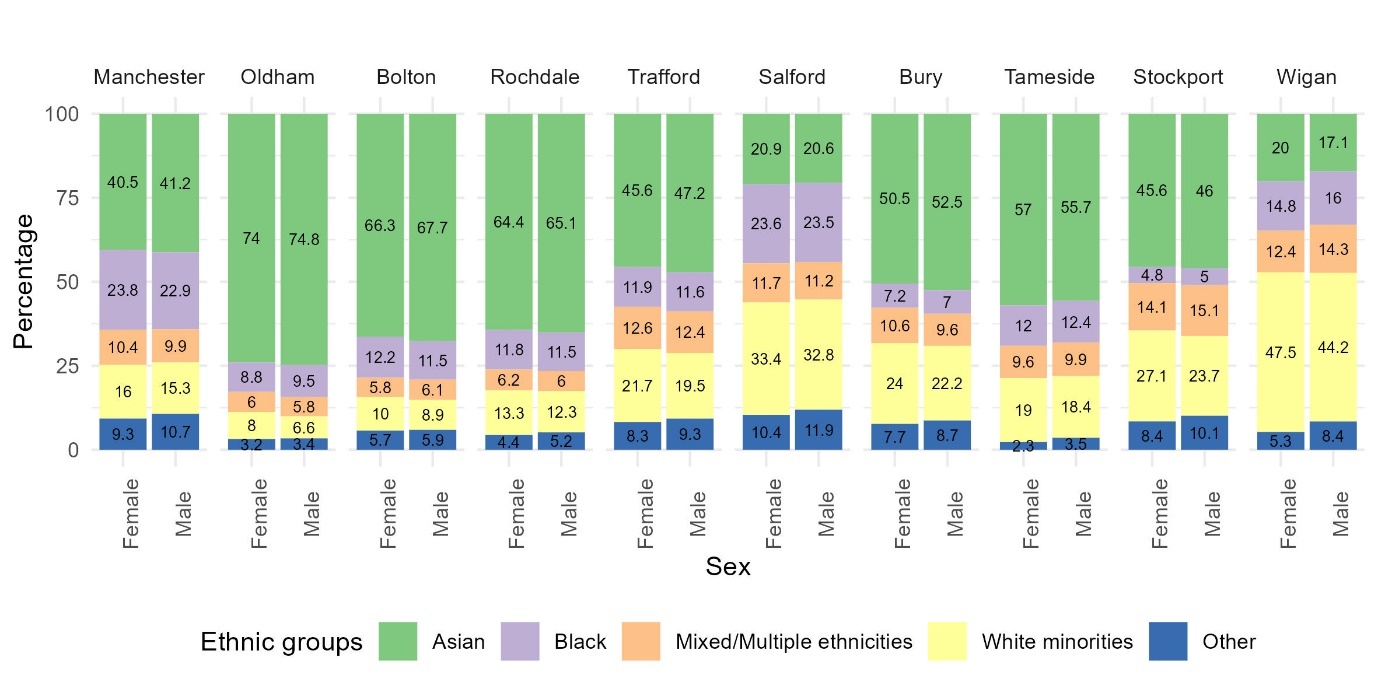


Supplementary Figure 6: Percentage of population by sex and broad minority ethnic groups based on the 2021 UK census categories in Greater Manchester boroughs, excluding the General Population (i.e. White British). The **Asian** group is composed of Bangladeshi, Chinese, Indian, Pakistani, and Other Asian. The **Black** ethnic group includes African, Caribbean, Other Black. The **Mixed or Multiple** ethnic groups refer to White and Asian, White and Black African, White and Black Caribbean, Other mixed or multiple ethnicities. **White minorities** are composed of Gypsy or Irish Traveller, Irish, Roma, Other White. **Other** ethnic group category involves Arab and any other ethnicity.


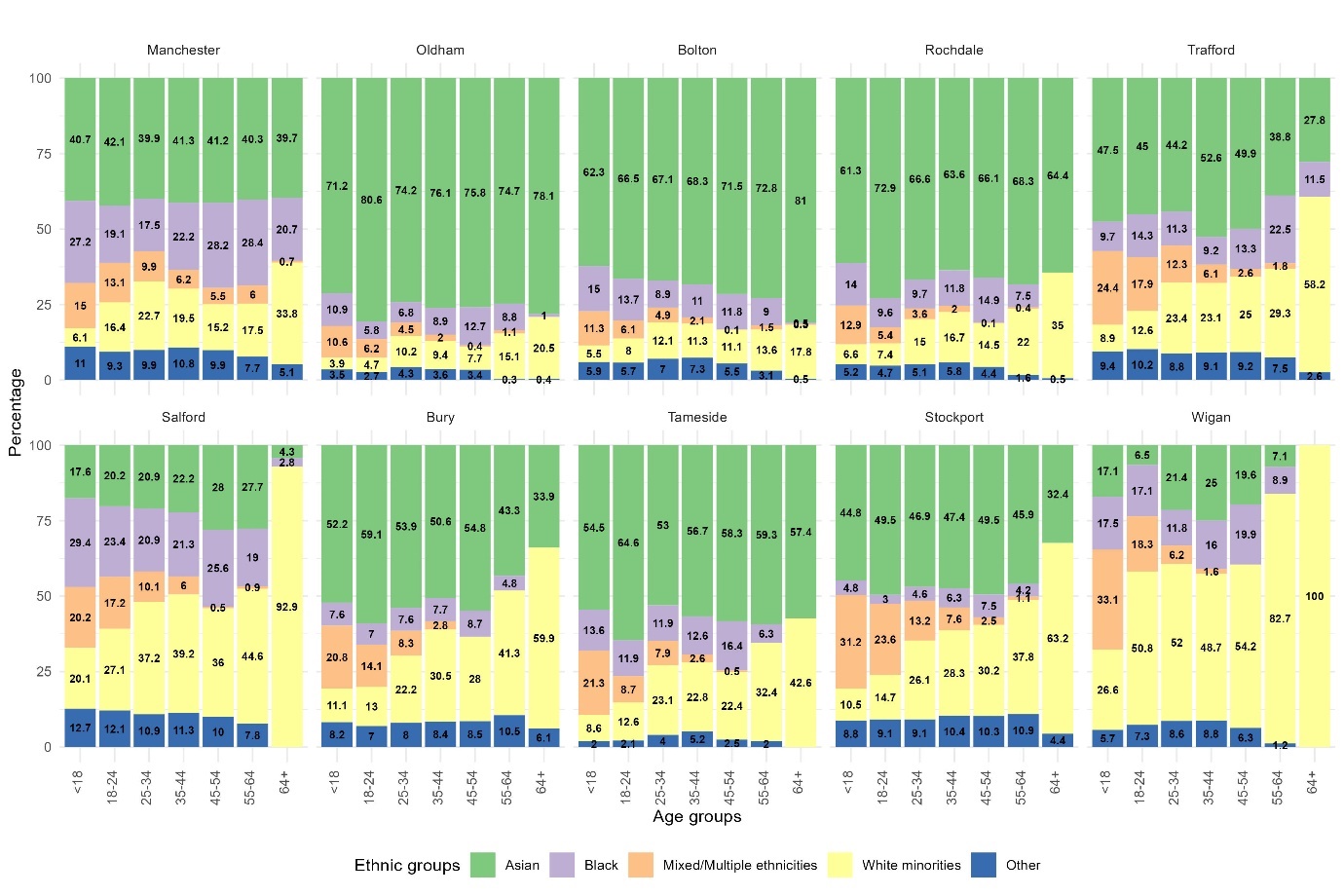


Supplementary Figure 7: Percentage of population by age groups and broad minority ethnic groups based on the 2021 UK census categories in Greater Manchester boroughs, excluding the General Population (i.e. White British). The **Asian** group is composed of Bangladeshi, Chinese, Indian, Pakistani, and Other Asian. The **Black** ethnic group includes African, Caribbean, Other Black. The **Mixed or Multiple** ethnic groups refer to White and Asian, White and Black African, White and Black Caribbean, Other mixed or multiple ethnicities. **White minorities** are composed of Gypsy or Irish Traveller, Irish, Roma, Other White. **Other** ethnic group category involves Arab and any other ethnicity.


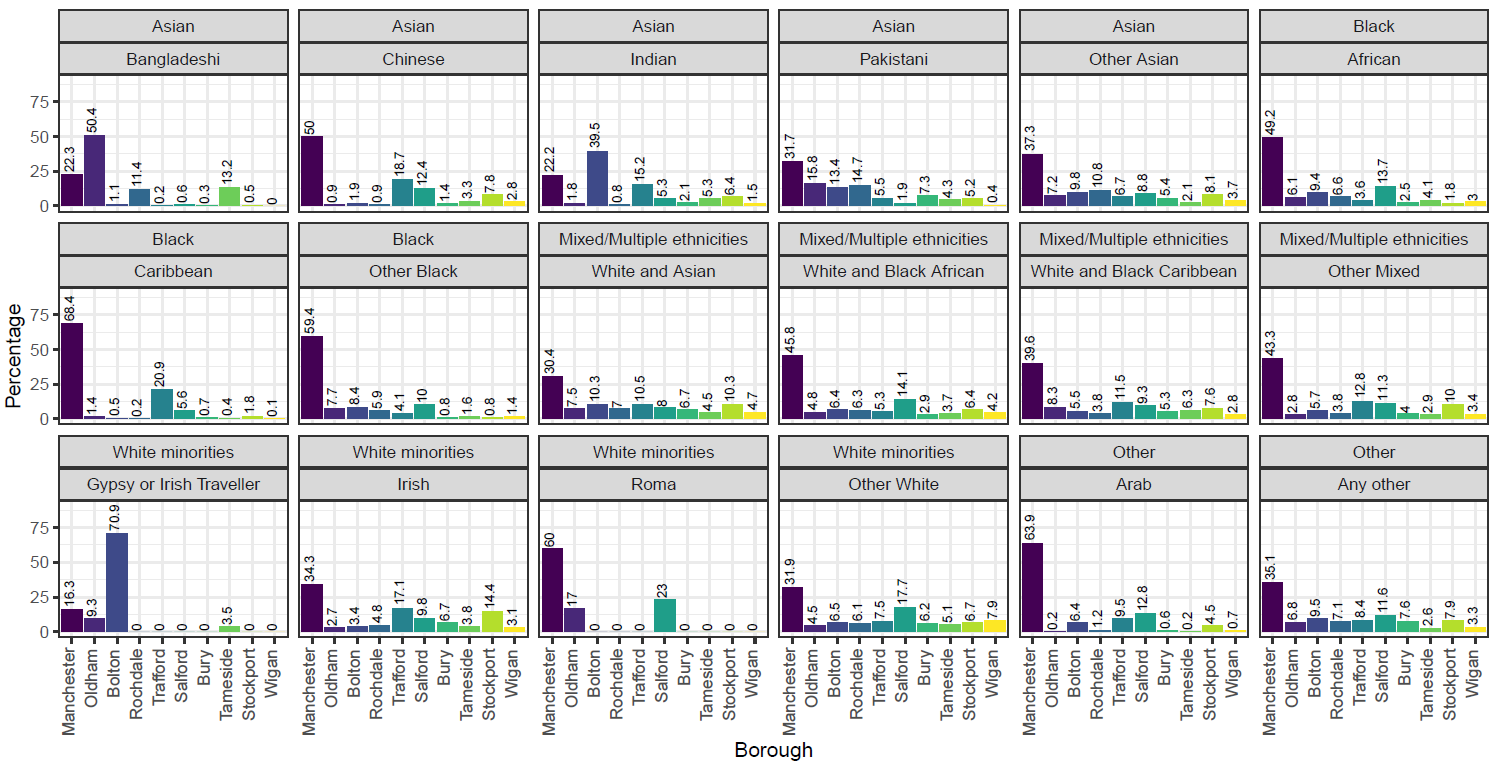


Supplementary Figure 8: Percentage of population by more detailed ethnic groups based on the 2021 UK census categories in Greater Manchester boroughs (summing to 100% across boroughs). The **Asian** group is composed of Bangladeshi, Chinese, Indian, Pakistani, and Other Asian. The **Black** ethnic group includes African, Caribbean, Other Black. The **Mixed or Multiple** ethnic groups refer to White and Asian, White and Black African, White and Black Caribbean, Other mixed or multiple ethnicities. **White minorities** are composed of Gypsy or Irish Traveller, Irish, Roma, Other White. **Other** ethnic group category involves Arab and any other ethnicity.

1. <https://www.ons.gov.uk/employmentandlabourmarket/peopleinwork/employmentandemployeetypes/methodologies/labourforcesurveyuserguidance>, Volume 10. [↑](#footnote-ref-2)
2. <https://www.ons.gov.uk/employmentandlabourmarket/peopleinwork/employmentandemployeetypes/articles/coronavirusanditsimpactonthelabourforcesurvey/2020-10-13> [↑](#footnote-ref-3)
3. <https://www.ons.gov.uk/employmentandlabourmarket/peopleinwork/employmentandemployeetypes/methodologies/labourmarketsurveyresearchandresultsoverview#what-you-need-to-know-about-this-release>. [↑](#footnote-ref-4)
4. <https://www.ons.gov.uk/peoplepopulationandcommunity/healthandsocialcare/healthandlifeexpectancies/methodologies/opinionsandlifestylesurveyqmi> [↑](#footnote-ref-5)
5. <https://www.ons.gov.uk/economy/economicoutputandproductivity/output/datasets/businessinsightsandimpactontheukeconomy> [↑](#footnote-ref-6)
6. <https://www.ons.gov.uk/economy/economicoutputandproductivity/output/datasets/businessinsightsandimpactonthesubnationalukeconomy> [↑](#footnote-ref-7)
7. <https://www.ons.gov.uk/economy/economicoutputandproductivity/output/datasets/businessimpactofcovid19surveybicsresults> [↑](#footnote-ref-8)
8. <https://www.ons.gov.uk/employmentandlabourmarket/peopleinwork/employmentandemployeetypes/methodologies/claimantcountqmi#:~:text=The%20Claimant%20Count%20is%20a,data%20from%20the%20benefits%20system.&text=The%20Claimant%20Count%20has%20undergone,Credit%20(UC)%20in%202013>. [↑](#footnote-ref-9)
